# Interactions between LHX3- and ISL1-family LIM-homeodomain transcription factors are conserved in *Caenorhabditis elegans*

**DOI:** 10.1038/s41598-017-04587-8

**Published:** 2017-07-04

**Authors:** Mugdha Bhati, Estelle Llamosas, David A. Jacques, Cy M. Jeffries, Siavoush Dastmalchi, Nina Ripin, Hannah R. Nicholas, Jacqueline M. Matthews

**Affiliations:** 1 School of Life and Environmental Sciences, University of Sydney, NSW, 2006 Australia; 2 Teva Pharmaceuticals Australia Pty Ltd, Macquarie Park, NSW, 2113 Australia; 3 School of Women’s and Children’s Health, University of New South Wales, NSW, Australia; 4 Department of Biology, ETH, Zurich, 8093 Switzerland; 5 iThree Institute, University of Technology, NSW, 2007 Australia; 6 European Molecular Biology Laboratory (EMBL) Hamburg Outstation, c/o DESY, Notkestrasse 85, 22607 Hamburg, Germany; 7 Biotechnology Research Center and School of Pharmacy, Tabritz Univeristy of Medical Science, Tabritz, Iran

## Abstract

LIM-Homeodomain (LIM-HD) transcription factors are highly conserved in animals where they are thought to act in a transcriptional ‘LIM code’ that specifies cell types, particularly in the central nervous system. In chick and mammals the interaction between two LIM-HD proteins, LHX3 and Islet1 (ISL1), is essential for the development of motor neurons. Using yeast two-hybrid analysis we showed that the *Caenorhabditis elegans* orthologs of LHX3 and ISL1, CEH-14 and LIM-7 can physically interact. Structural characterisation of a complex comprising the LIM domains from CEH-14 and a LIM-interaction domain from LIM-7 showed that these nematode proteins assemble to form a structure that closely resembles that of their vertebrate counterparts. However, mutagenic analysis across the interface indicates some differences in the mechanisms of binding. We also demonstrate, using fluorescent reporter constructs, that the two *C. elegans* proteins are co-expressed in a small subset of neurons. These data show that the propensity for LHX3 and Islet proteins to interact is conserved from *C. elegans* to mammals, raising the possibility that orthologous cell specific LIM-HD-containing transcription factor complexes play similar roles in the development of neuronal cells across diverse species.

## Introduction

**Figure 1:**
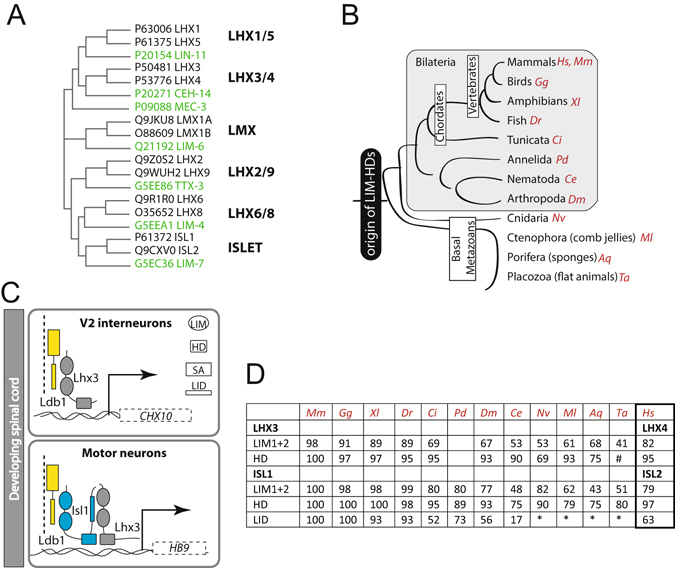
Islet- and LHX3-family LIM-HD proteins and interactors. (**A**) Simple phylogenetic tree diagram of LIM-HD proteins from mouse (black) and *C. elegans* (green) illustrating the LIM-HD families; Uniprot accession codes are provided. Branches and relationships are derived from analysis of these proteins using CLUSTAL OMEGA and CLUSTAL PHYLOGENY. Distances are not to scale. (**B**) Schematic of evolutionary relationships in metazoans. *Hs – Homo sapiens; Ms – Mus musculus; Gg – Gallus gallus, Xl – Xenopus laevis, Dr – Danio rerio, Ci – Ciona intestinalis, Pd - Platynereis dumerilii*, *Ce -Caenorhabditis elegans, Dm – Drosophila melanogasta, Nv - Nematostella vectensis, Ml - Mnemiopsis leidyi, Aq- Amphimedon queenslandica, Ta - Trichoplax adhaerens*. The branchpoints between some basal metazoans are controversial^70, 71^ and not indicated here. (**C**) Distinct transcriptional complexes drive different transcription programs in adjacent cell types in the developing ventral spinal cord in vertebrates. (**D**) Sequence identity (% compared to *Hs* proteins) between domains in metazoan LHX3 and ISL1 proteins. No domain identified (*), substantially truncated domain identified (^#^). No *Pd*LHX3 gene was found.

LIM-containing proteins are commonly found in eukaryotes of all types, but LIM-homeodomain (LIM-HD) transcription factors are unique to and highly conserved in animals. The proteins are characterised by two closely-spaced LIM domains (zinc fingers that mediate protein-protein interactions) at or near their N-termini, a central homeodomain (which binds DNA) and a C-terminal domain, the functions of which are usually unknown. There are six subfamilies of LIM-HD proteins (Fig. 1A). Vertebrates have two representatives from each subfamily (except teleost fish, which have up to four representatives from each subfamily), whereas most invertebrates have only one gene from each, or lack representation from one or more subfamilies^1^. Basal metazoans, which have simpler body plans, also contain genes for LIM-HD proteins, but none have been identified in plants or unicellular organisms suggesting that this family of proteins expanded and diversified early in metazoan evolution^2, 3^ (Fig. 1B). The contribution of LIM-HD proteins to cell specification and tissue patterning has been well studied in vertebrate neuronal development. Early observations that different combinations of LIM-HD proteins were expressed in different neurons^4, 5^, led to suggestions that a combinatorial transcriptional code (the “LIM-code”) involving these proteins was responsible for neuronal subtype specification^6^.

The best characterised example of the LIM code relates to the developing ventral spinal cord in vertebrates. It involves LIM-HD factors LIM homeobox protein 3 (LHX3) and Islet 1 (ISL1). The expression of LHX3 in the absence of ISL1 in one population of post-mitotic cells results in V2 interneuron formation, whereas the expression of both LHX3 and ISL1 in an adjacent set of cells leads to motor neuron formation^7^. The alternate differentiation outcomes result from the formation of cell-specific transcription complexes. In developing V2 interneurons LHX3 binds to LIM-domain binding protein 1 (LDB1), which is an essential cofactor for LIM-HD proteins^8^, and this binary complex binds to LHX3 recognition elements on DNA, including those in the promoter region of *Chx10*, a V2 interneuron marker^9^ (Fig. 1C). In developing motor neurons ISL1 binds directly to LDB1, while LHX3 binds instead to ISL1. This ternary complex binds to ISL1/LHX3 recognition sites, including those in the promoter region of *Hb9*, which is a marker of motor neurons (Fig. 1C). The interaction between LHX3 and ISL1, first noted by Jurata and colleagues^10^, is mediated by the tandem LIM domains of LHX3 and a ~30-residue region in the C-terminal domain of ISL1 that was designated as the LHX3-binding domain in ISL1^11^, but is hereafter referred to as a LIM interaction domain (LID). The structures of LHX3 in complex with each of ISL1 and LDB1 revealed that both proteins bind LHX3 in the same manner, despite considerable sequence variation in the interaction domains^11^.

Members of the same LIM-HD protein subfamily are often expressed in the same neural cell type. For example, ISL1/ISL2 and LHX3/LHX4 pairs are all expressed in developing motor neurons. Interactions between ISL1/LHX4, ISL2/LHX3 and ISL2/LHX4 have all been detected *in vivo* ^10, 12^, and the structures of the complexes are highly similar^11, 12^. Equivalent complexes are likely to exist in zebrafish and Drosophila, although the molecular details and functional roles of such complexes are less well documented^7, 10, 11, 13–16^. To determine if LHX3/ISL1-type interactions are widely conserved in metazoans, we focussed on the more divergent but highly characterised nematode *Caenorhabditis elegans*. These nematodes have seven LIM-HD proteins (one member from six subfamilies, and an additional member that is most closely related to the LHX3/LHX4 and LHX1/LHX5 families^17, 18^, Fig. 1A), all of which are expressed in neurons^18–25^. Early observations of expression patterns of LIM-HD proteins found little or no overlapping expression in *C. elegans*, suggesting that interactions between LIM-HD proteins might not be an evolutionarily conserved feature^18^. The *C. elegans* orthologs of ISL1 and LHX3 are LIM-7 and CEH-14, respectively. We hypothesised that these two LIM-HD proteins can interact to form cell-specific transcription complexes like their vertebrate counterparts. We identified an ISL1_LID_-like domain in LIM-7 and tested the ability of this domain to interact with CEH-14. The LIM interaction domain of LIM-7 (LIM-7_LID_) interacts with the tandem LIM domains of CEH-14 (CEH-14_LIM1+2_) in a similar fashion to ISL1_LID_ contacting LHX3_LIM1+2_. Biophysical characterisation of a tethered complex of LIM-7_LID_ with CEH-14_LIM1+2_, revealed that this complex is identical to the mouse counterpart and to other LIM/LID transcriptional assemblies that we have previously reported. We also demonstrate that the cellular expression patterns of fluorescently tagged reporter constructs of these genes, *lim-7::mcherry* and *ceh-14::gfp*, overlap in a restricted number of head and tail neurons. These data suggest that LHX3/ISL1 family interactions also exist in nematodes and have been conserved from early animal evolution.

## Results

### Identification of the LIM interaction domain of LIM-7

**Figure 2:**
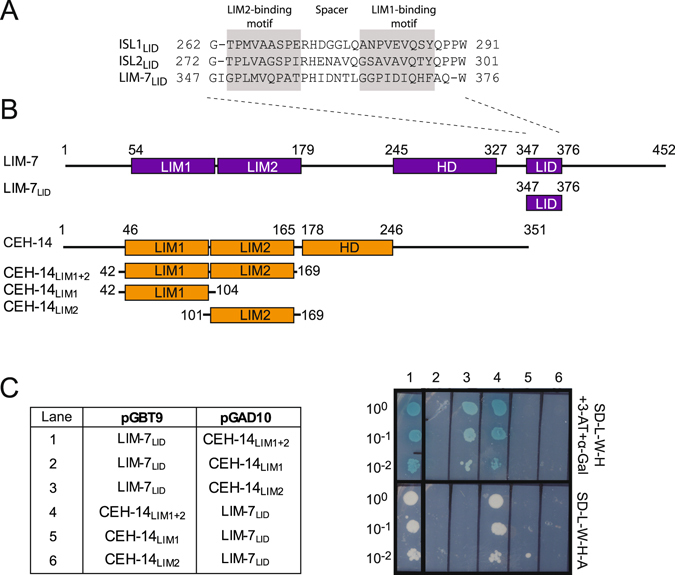
Islet- and LHX3-family LIM-HD proteins from *C. elegans* interact. (**A**) Manual alignment of experimentally determined LIM-interaction domains from mouse ISL1 and ISL2 and a predicted LID from LIM-7. The LIM1 and LIM2 binding motifs as previously identified for ISL1 and ISL2 are indicated; the spacer is the sequence between the binding motifs. (**B**) Schematics of LIM-7 and CEH-14 and constructs used in yeast two-hybrid (Y2H) assays showing the LIM1 and LIM2, homeodomain (HD) and predicted LIM interaction domain (LID). (**C**) Y2H data indicate a direct interaction between LIM-7_LID_ and CEH-14_LIM1+2_. The original images for the yeast plates, including additional controls are shown in Supplemental data Figure S1.

A comparison of sequences of ISL and LHX3 family proteins from a range of metazoan species that represent different evolutionary branches (Supplemental Data 1) indicates that there is high conservation of HDs and LIM domains, but the ISL_LID_ is less conserved (Fig. 1D). A BLAST search using a 31-residue sequence of murine ISL1_LID_ detected similar domains in ISL-proteins in vertebrates, arthropods and chordata, but failed to identify any similar sequences within LIM-7. However, a manual alignment of the C-terminal regions of ISL1 and LIM-7 that used the spacing of the HD and LID domains as well as the structurally conserved LIM-binding motifs in the LID domains^26^ as a guide, revealed a putative LIM interaction domain encompassing residues 347–376 of LIM-7 (LIM-7_LID_; Fig. 2A). This region shares only 17% sequence identity with mammalian ISL1_LID_ compared with higher levels of identity in the LIM domain (48%) and HD (75%) regions. Similar sequences could not be identified in basal metazoans that contain both ISL- and LHX3-like proteins.

We predicted that an interaction between CEH-14 and LIM-7 would occur via the LIM domains of CEH-14 (CEH-14_LIM1+2_) and the putative LIM-7_LID_, by analogy with the previously-observed interactions between LHX3/4 and ISL1/2 proteins^11, 12^. We used yeast two-hybrid (Y2H) analysis to investigate whether CEH-14 and LIM-7 could physically interact. Control experiments, in which the full length LIM-HD proteins in pGBT9 plasmids were paired with ‘empty’ pGAD10 vectors, gave rise to yeast growth indicating autoactivation of the reporter genes as described previously^27^. This is not unexpected due to the presence of the DNA-binding homeodomains and possible activation domains in the C-terminal domains for these proteins. However, by focussing on smaller constructs (Fig. 2B) in the assays, an interaction between LIM-7_LID_ and CEH-14_LIM1+2_ was observed as evidenced by yeast growth on moderate (-L-W-H + 3-AT) and high (-L-W-H-A) stringency media (Fig. 2C and Supplemental data Figure S2). LIM-7_LID_ was also tested for binding to each of the individual LIM domains of CEH-14, CEH-14_LIM1_ and CEH-14_LIM2_. In those experiments, LIM-7_LID_ could not bind with CEH-14_LIM1_ alone but could bind weakly (yeast growth under moderate but not high affinity selection conditions) to CEH-14_LIM2_, only when the latter was expressed from a pGAD10 plasmid. Note that it is not unusual to see apparent differences in strength of binding for Y2H interactions with the bait and prey proteins in alternate vector combination as was seen here; differences in yeast growth may originate from differences in protein stability in the different constructs^11, 12^. Thus, whereas both LIM domains from CEH-14 are required for high affinity binding to LIM-7, CEH-14_LIM2_ is able to bind with LIM-7 independently.

### Biophysical analysis of a CEH-14/LIM-7 complex

**Figure 3:**
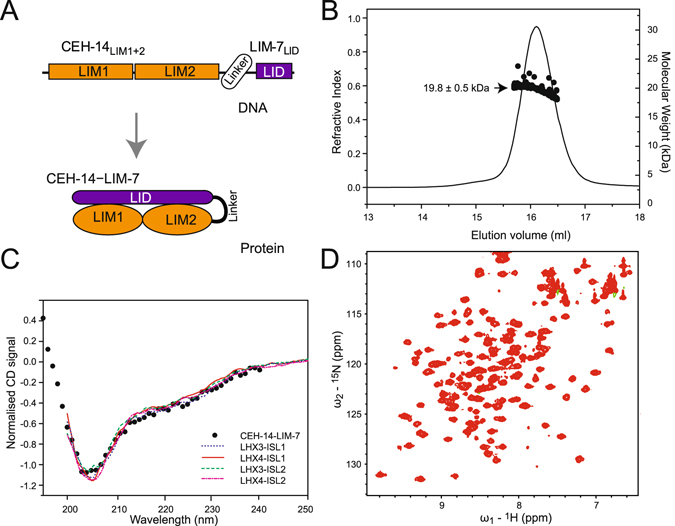
A ‘tethered’ CEH-14–LIM-7 complex. (**A**) Schematic of the CEH-14–LIM-7 tethered complex. Based on data from similar complexes, introducing a tether between the LIM1+2 domains from CEH-14 and the LID from LIM-7 was expected to stabilise the complex and facilitate structural characterisation. This engineering approach takes advantage of the close positioning of C-and N-termini in the native complexes. (**B**) SEC-MALLS data for CEH-14–LIM-7 in 20 mM Tris, pH 8.0, 150 mM NaCl, 1 mM DTT at 0.5 ml/min at 25 °C (black line, refractive index profile; black dots, MW distribution). (**C**) Far UV-CD profile of CEH-14–LIM-7 (5 μM black dots) in 10 mM Tris-HCl pH 8.5, 150 mM NaF, 0.5 mM TCEP at 20 °C, compared to the spectra of related LHX3/4–ISL1/2 tethered complexes as indicated. Data for those complexes were published previously^12^. (**D**) ^1^H-^15^N HSQC spectrum of CEH-14–LIM-7 (37 μM) in 20 mM HEPES pH 7.0, 1 mM DTT at 25 °C recorded at 600-MHz.

Having obtained evidence of a direct interaction between LIM-7 and CEH-14, we set out to examine whether the association of these proteins is structurally analogous to that of LHX3 and ISL1 using biophysical analyses. When recombinant forms of LIM domains from LIM-HD proteins are expressed in bacteria, they tend to aggregate and/or be expressed in inclusion bodies, but they can be stabilised through tethering to an interaction partner such as LDB1_LID_ or ISL1/2_LID_^28–31^. The same approach was used here such that the C-terminus of the LIM domains from CEH-14 was tethered to the N-terminus of the LIM-7_LID_ using an 11-residue Gly/Ser linker to form CEH-14–LIM-7 (Fig. 3A). This construct expressed as a predominantly soluble protein in *E. coli*. SEC-MALLS data showed that purified CEH-14–LIM-7 is largely monomeric at high micromolar concentrations (200 μM loading concentration, ~25 μM at the maximum of the monomer elution peak based on refractive index), with an average molecular weight of 19.8 ± 0.5 kDa (Fig. 3B) which is in close agreement with the calculated molecular weight (19.4 kDa) based on amino acid sequence composition.

### The solution structure of a CEH-14/LIM-7 complex

**Figure 4:**
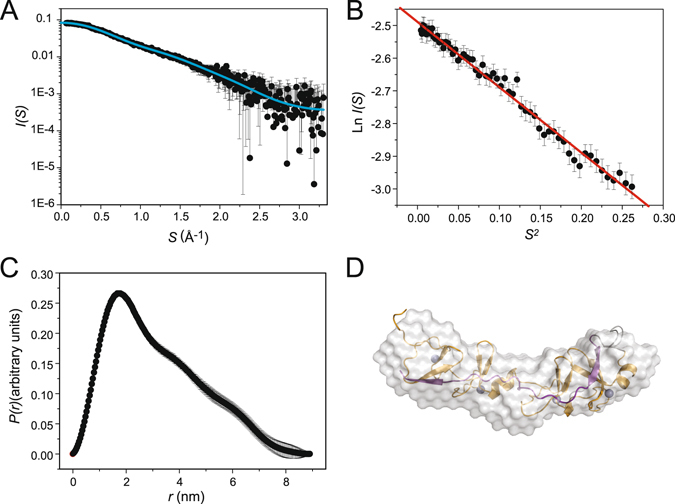
SAXS analysis for CEH-14–LIM-7. (**A**) Buffer-corrected, desmeared SAXS scattering curve of CEH-14–LIM-7 (circles; 5.2 mg mL^−1^) in 20 mM Tris pH 8.0, 150 mM NaCl, 1 mM TCEP, overlaid with the theoretical scattering profiles of an homology model of CEH-14–LIM-7 based on the template of the LHX3-ISL1 crystal structure (1RGT Chain B; cyan line) calculated by CRYSOL. (**B**) Guinier plot showing linearity of low Q data. (**C**) The pairwise distribution *P*(*r*) profile. (**D**) Shape restoration model of CEH-14–LIM-7 (white surface) superimposed with an homology model based on the template of the LHX3-ISL1 crystal structure (orange and purple; zinc ions are shown as grey spheres).

Given that the sequences of LIM-7_LID_ are quite different from those of ISL1/2_LID_ we sought to determine the structure of the CEH-14/LIM-7 complex. The far-UV circular dichroism spectrum of CEH-14–LIM-7 is characteristic of a folded protein, and closely resembles the spectra of related LHX3/4-ISL1/2 complexes (Fig. 2C)^12^. Various attempts were made to determine the high resolution structure of CEH-14–LIM-7. ^1^H-^15^N-HSQC data showed that the protein was well folded (Fig. 3D), but limited sample solubility meant that it was not feasible to use NMR methods to determine the solution structure of CEH-14–LIM-7 (data not shown). Although crystals of the tethered complex formed under several conditions, they diffracted only to low resolution and/or displayed high levels of anisotropy, preventing structure determination. Fortunately, however, the low resolution structure of the complex could be derived using small angle X-ray scattering (SAXS). When combining the structural parameters extracted from the SAXS data (Fig. 4 and Table 1) with *a priori* shape classification and subsequent structural modelling, the SAXS results show that CEH-14–LIM-7 adopts an overall extended conformation that is structurally very similar to the LHX3-ISL1 homologue^12^. The SAXS-based molecular weight (*M*_*r*_) estimates of ~17–20 kDa, assessed from concentration-dependent and concentration-independent methods^32–34^, lie close to the expected value of 19.4 kDa for a monomeric complex and are commensurate with the experimental *M*_*r*_ results obtained from MALLS (19.8 kDa). These *M*_*r*_ values, an obtained Porod volume of ~26 nm^3^ (expected dry volume = 23 nm^3^) and the linear Guinier plot of the data at very-low angles (ln*I*(*s*) vs *s* ^2^, *s* ^2^ < 0.26 nm^−2^, *R* ^2^ = 0.985; Fig. 4B)^35^, indicate that the complex is homogeneous and unaffected by self-association/oligomerisation, aggregation or repulsive interparticle interference effects. In summary, the SAXS data indicate that CEH-14–LIM-7 is a monomeric tethered complex in solution.

**Table 1:** Small angle scattering parameters for CEH-14–LIM-7.

Data-collection parameters	
Instrument	SAXSess (Anton Paar)
Beam geometry	10 mm slit
X-ray wavelength (nm)	0.1542
Measured *s*-range (nm^−1^)	0.07–4.0
Shannon-channel limited *s*-range (nm^−1^)^a^	0.07–3.3
Exposure time (min)	4 × 15 min
Protein Concentration (mg ml^−1^)^b^	5.2 (±0.3)
Temperature (°C)	10
Structural parameters	
*I*(0) (cm^−1^) [from *p*(*r*)]	0.0833 ± 0.002
Real-space *R*_g_ (nm) [from *p*(*r*)]	2.5 ± 0.09
*I*(0) (cm^−1^) (from Guinier)	0.083 ± 0.0002
*R*_g_ (nm) (from Guinier)	2.4 ± 0.03
*D*_max_ (nm)	8.9
Porod volume estimate (V_p_, nm^3^)	25.6
Molecular-mass determination	
Partial specific volume (cm^3^ g^−1^)^c^	0.718
Contrast (Δρ × 10^10^ cm^−2^)^c^	3.345
Molecular mass *M*_r_, kDa [from *I*(0)]^d^	16.7 ± 0.9
Molecular mass *M*_r_, kDa [from SAXSMOW]^d^	20
Molecular mass *M*_r_, kDa [from Vc]^d^	18
Expected monomeric *M*_r_ calculated from sequence, kDa	19.4
Software employed	
Primary data reduction	*SAXSquant* 1D
Data processing	*PrimusQT/GNOM*
*Ab initio* analysis	*DAMMIF*
Spatial averaging and resolution estimates	*DAMAVER/SASRES*
Computation of model intensities	*CRYSOL*

^a^The information content of the scattering data and corresponding *s*-range were evaluated using SHANUM^62^. ^b^The protein concentration was determined at A_280 nm_ using the extinction coefficient calculated by ProtParam^59^ from the primary amino acid sequence. ^c^Obtained from the *Contrast* module of MULCH^61^. ^d^Molecular mass estimates were determined from *I*(0) and protein concentration (*M*_r_ = *I*(0)N_A_/[protein](Δρυ)^2^) as well as concentration-independent methods (SAXSMOW^32^ and the volume of correlation, Vc^33^).

A qualitative assessment of the Kratky plot obtained from the SAXS data (*I*(*s*)*s* ^2^ vs *s*) indicates that the protein is mainly folded^36^, while the model-independent shape classification and the shape topology determined from the data using automated shape-categorization and ambiguity assessments^37^ suggest that the complex is structurally anisotropic and extended (Supplemental data Figure S3). The conclusion that CEH-14–LIM-7 forms an extended particle is borne out in the resulting scattering-pair distance distribution (*p*(*r*) vs *r* profile) that shows a skewed distribution of real-space vector lengths for *r* > 1.8 nm which extends to a maximum particle dimension, *D*_*max*_, of ~9 nm with a radius of gyration, *R*_*g*_, of 2.5 nm (Fig. 4C).

The reconstruction of the low-resolution shape of the CEH-14–LIM-7 complex was calculated using *ab initio* dummy atom bead modelling^38^. Ten individual models were generated that each fit the experimental data (*χ* ^2^ = 0.3). Although the *χ* ^2^ discrepancy of the individual model-fits to the data is somewhat low, likely due to the misspecification of experimental errors, no statistically significant systematic deviations between the data and the fits were identified using variance/co-variance analysis (Correlation Map, or CorMap, *p* > 0.01)^39^. The spatial alignment and averaging of the individual models produces a mean normalised spatial discrepancy of 0.6^40^, indicating spatial consistency between the ten reconstructions, with an ensemble resolution estimate of 2.8 nm^41^. The final averaged representation of the low-resolution structure of CEH-14–LIM-7 (corrected for volume and spatial occupancy) is presented in Fig. 4D and shows that the complex adopts a subtly bent and extended conformation in solution with approximate dimensions of ~3 × 2 × 9 nm. This global conformation is very similar to that previously observed for the neuronal-type specification complex LHX3-ISL1^12^. Indeed, the scattering profile of LHX3-ISL1 calculated from the X-ray crystal structure (PDB: 2RGT chain B) fits the CEH-14–LIM-7 SAXS data surprisingly well (*χ* ^2^ = 0.3; CorMap *p* = 0.025; Supplementary data Figure S4A) and was thus used as a template to develop an atomistic homology model of CEH-14–LIM-7 (see below) that fits that solution scattering data (*χ* ^2^ = 0.3; CorMap *p* = 0.05). Both the CEH-14–LIM-7 homology model and the LHX3–ISL1 X-ray crystal structure spatially superpose very well into the low-resolution *ab initio* bead model of the complex (Fig. 4D and Supplemental data Figure S4B).

### Binding determinants on CEH-14_LIM1+2_ and LIM-7_LID_

Several series of LIM-7_LID_ mutants were generated and tested for binding with CEH-14 in yeast two-hybrid assays in order to identify which residues from LIM-7_LID_ are critical for binding to CEH-14 (Table 2). Initially the LIM-7 residues P350, L351, M352 and V353, were mutated to alanine as single or double mutations, as equivalent residues have previously been shown to be important for the interaction between LHX3 and ISL1^11^. However, none of these mutations had any significant effect on the interaction with CEH-14_LIM1+2_ in this assay. Thus, an alanine scan covering the entire LIM-7_LID_ peptide was used to probe for key regions of binding. Sets of three consecutive residues were mutated to alanine (or glycine, if the wildtype residue was alanine) and tested for binding. Only one mutant LIM-7(H359A/I360A/D361A) completely abolished the interaction of the peptide with CEH-14_LIM1+2_ under both moderate and strong selection conditions. Two other mutants, LIM-7(P350A/L351A/M352A) and LIM-7(A374G/Q375A/W376A), showed some evidence of a reduced interaction as yeast growth was observed only under moderate (but not high) affinity selection conditions. Residues H359, I360 or D361 were individually targeted for mutation, with no major effect on binding, but when mutated in combination with the P350A/L351A/M352A triple mutant, each mutation was sufficient to abolish binding in this assay. Overall, these data indicate that residues H359, I360 and D361 in LIM-7 are the most important for the interaction, whereas residues within P350-M352 and A374-W376 also play a more modest role.

**Table 2:** Yeast two-hybrid mutagenic screens for CEH-14 and LIM-7 interactions.

pGBT9	*Sequence*	pGAD10
*Construct*		
LIM-7_LID_		**CEH-14**_**LIM1+2**_
WT LIM-7_LID_	GIGPLMVQPATPHIDNTLGGPIDIQHFAQW	+++/+++
P350A	GIG**A**LMVQPATPHIDNTLGGPIDIQHFAQW	+++/+++
L351A	GIGP**A**MVQPATPHIDNTLGGPIDIQHFAQW	+++/+++
M352A	GIGPL**A**VQPATPHIDNTLGGPIDIQHFAQW	+++/+++
V353A	GIGPLM**A**QPATPHIDNTLGGPIDIQHFAQW	+++/+++
P350A/L351A	GIG**AA**MVQPATPHIDNTLGGPIDIQHFAQW	+++/++
L351A/M352A	GIGP**AA**VQPATPHIDNTLGGPIDIQHFAQW	+++/+++
G347A/I348A/G349A	**AAA**PLMVQPATPHIDNTLGGPIDIQHFAQW	+++/+++
P350A/L351A/M352A	GIG**AAA**VQPATPHIDNTLGGPIDIQHFAQW	+++/−
V353A/Q354A/P355A	GIGPLM**AAA**ATPHIDNTLGGPIDIQHFAQW	+++/+++
A356G/T357A/P358A	GIGPLMVQP**GAA**HIDNTLGGPIDIQHFAQW	+++/+++
H359A/I360A/D361A	GIGPLMVQPATP**AAA**NTLGGPIDIQHFAQW	−/−
N362A/T363A/L364A	GIGPLMVQPATPHID**AAA**GGPIDIQHFAQW	+++/+++
G365A/G366A/P367A	GIGPLMVQPATPHIDNTL**AAA**IDIQHFAQW	+++/+++
I368A/D369A/I370A	GIGPLMVQPATPHIDNTLGGP**AAA**QHFAQW	+++/+++
Q371A/H372A/F373A	GIGPLMVQPATPHIDNTLGGPIDI**AAA**AQW	+++/+++
A374G/Q375A/W376A	GIGPLMVQPATPHIDNTLGGPIDIQHF**GAA**	+++/−
H359A	GIGPLMVQPATP**A**IDNTLGGPIDIQHFAQW	+++/+++
I360A	GIGPLMVQPATPH**A**DNTLGGPIDIQHFAQW	+++/+++
D361A	GIGPLMVQPATPHI**A**NTLGGPIDIQHFAQW	+++/+++
P350A/L351A/M352A/H359A	GIG**AAA**VQPATP**A**IDNTLGGPIDIQHFAQW	−/−
P350A/L351A/M352A/I360A	GIG**AAA**VQPATPH**A**DNTLGGPIDIQHFAQW	−/−
P350A/L351A/M352A/D361A	GIG**AAA**VQPATPHI**A**NTLGGPIDIQHFAQW	−/−

Y2H assay summary for alanine mutagenic screening of LIM-7_LID_ against CEH-14_LIM1+2_. Results are reported as yeast growth on moderate (SD^-H-L-W+1mM 3-AT^)/strong (SD^-H-L-W-A^) selective media. Yeast growth is represented as either ‘+++’, ‘++’ or ‘+’ for robust growth at all three dilution points (10°, 10^−1^, 10^−2^), two dilution points (10°, 10^−1^) or only on the first dilution point (10^0^), respectively. ‘−’ represents no detectable yeast growth. LIM-7_LID_ and CEH-14_LIM1+2_ constructs were in the vectors pGBT9 and pGAD10, respectively.

### Homology Modelling of CEH-14–LIM-7

**Figure 5:**
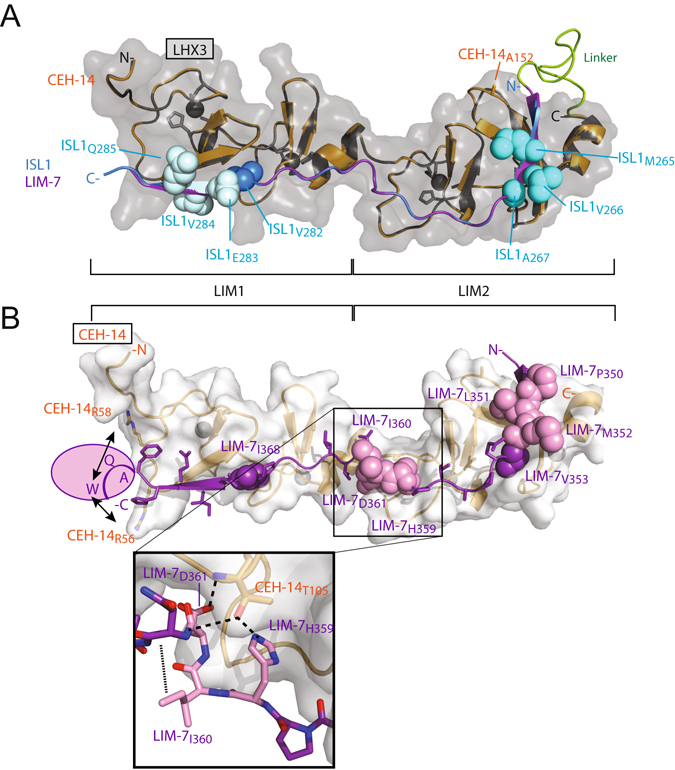
Conserved and unusual features of LHX3-Islet family LIM-HD interactions in *C. elegans* proteins. (**A**) Overlay of the LHX3_LIM1+2_ (black ribbon/transparent grey surface representation)–ISL1_LID_(cyan) crystal structure (1RGT Chain B) and a simple homology model of CEH-14(orange)–LIM-7(purple). The N- and C-termini from the domains in LHX3 and ISL1 are indicated. A small insertion in CEH-14 (residue A152) is likely to have little effect apart from a short β-hairpin extension. The binding hotspots for ISL1 for LHX3 and LHX4 as determined by alanine scanning mutagenesis and Y2H are shown (cyan spheres), an additional hotspot for ISL2 binding to LHX3 and LHX4 is also indicated (light cyan spheres) and a conserved residue (ISL1_V282_) that is buried between the two zinc binding modules is indicated (blue spheres). (**B**) Predicted binding features of CEH-14 (orange ribbon; white surface representation) and LIM-7 (purple ribbon and stick representation). The binding hotspots are indicated (pink circles/spheres). LIM-7_V353/I368_ (purple spheres) are buried between the two zinc binding modules of each LIM domain in CEH-14. Inset shows possible stabilising interactions within the main hotspot with residues in stick representation. Possible stabilising interactions between LIM-7_W376_ and CEH-14_R56/R58_ are indicated. Nitrogen atoms (blue) and oxygen atoms (red) are shown where appropriate.

Given that the far-UV CD and SAXS data for LHX3_LIM1+2_–ISL1_LID_ and CEH-14_LIM1+2_–LIM-7_LID_ complexes indicate that the structures are very similar, we generated a simple homology model of the *C. elegans* complex using SwissModel. As with homology models in general this model closely resembles the template (Fig. 5A) and may not reflect minor differences in conformation between the two complexes. Per residue QMEAN scores from SwissModel indicate that the model is of higher quality around the core part of each LIM domain-binding motif interaction module, and is less likely to be accurate in the Gly/Ser linker and ends of the domains (which were not present in the X-ray coordinates), loops, and around the spacer between the LIM-binding motifs (Supplemental Data 5). However, it fits the acquired SAXS data from the CEH-14_LIM1+2_–LIM-7_LID_ complex in solution (Fig. 4D) and allows a reasonable physical interpretation of the mutational data above. LIM domains share a highly conserved structure with some variations in the spacing between zinc-ligating residues, which generally just change loop lengths. For the LIM domains from CEH-14 and LHX3, the spacing of the zinc-coordinating residues are identical, apart from a single loop where CEH-14 has an extra residue (CEH-14_A152_) in the bend of the final β-hairpin of the LIM2 domain, which should have a very minor effect on the local structure (Fig. 5A). In terms of the LIDs from LIM-7 and ISL1, the sequence identity is low, and it is not easy to confidently predict binding registers for LIM-LID interactions^43, 44^. However, the SWISS-MODEL prediction was identical to our manual alignment (Fig. 2A), and indicates that the most important residues for binding (H359–D361), as identified by mutagenesis, lie in the spacer region between the two predicted LIM-binding motifs (Fig. 5B). In the CEH-14–LIM-7 model and the LHX3–ISL1 structure, equivalent residues LIM-7_H359_ and ISL1_H272_ sit in pockets in the LIM2 domain of the partner protein such that the imidazole groups of those residues make complementary interactions with the sidechain Oγ atoms of CEH-14_T105_ and LHX3_T91_, respectively (Fig. 5B). The sidechain of LIM-7_I360_ appears to make intramolecular hydrophobic contacts with the backbone of LIM-7_N362_, while the sidechain of LIM-7_D361_ appears to sit between the two LIM domains of CEH-14, and could make favourable intermolecular interactions with the imidazole sidechain of LIM-7_H359_ and backbone N of CEH-14_T105_. Of the two other regions in LIM-1_LID_ that were identified as making moderate contributions to binding, one (P350/L351/M352) lies in the putative LIM2-binding motif and is similar to the main binding-hotspots previously identified for ISL1/2 binding to LHX3/4^11, 12^; all three sidechains in this cluster appear to make hydrophobic contacts with the surface of the LIM domains. In contrast, the second binding hotspot, A374G/Q375A/W376A, lies outside of the structured regions. It is possible that mutation of A374 to glycine introduces too much flexibility, destabilising the peptide, and/or that W376 makes additional hydrophobic or other interactions with CEH-14 (e.g., π-cation interactions with CEH-14_R56/R58_). Another characteristic feature of LIM-LID interactions^26^ that is conserved in this model is the burial of the LIM-7_V353_ and LIM-7_I368_ sidechains in between the two zinc-binding modules in each LIM domain of CEH-14. Note that although these features are structurally conserved, mutation of these buried residues can have surprisingly little effect, possibly due to plasticity in the hydrophobic pockets^31, 44^.

### *ceh-14* and *lim-*7 expression patterns

**Figure 6:**
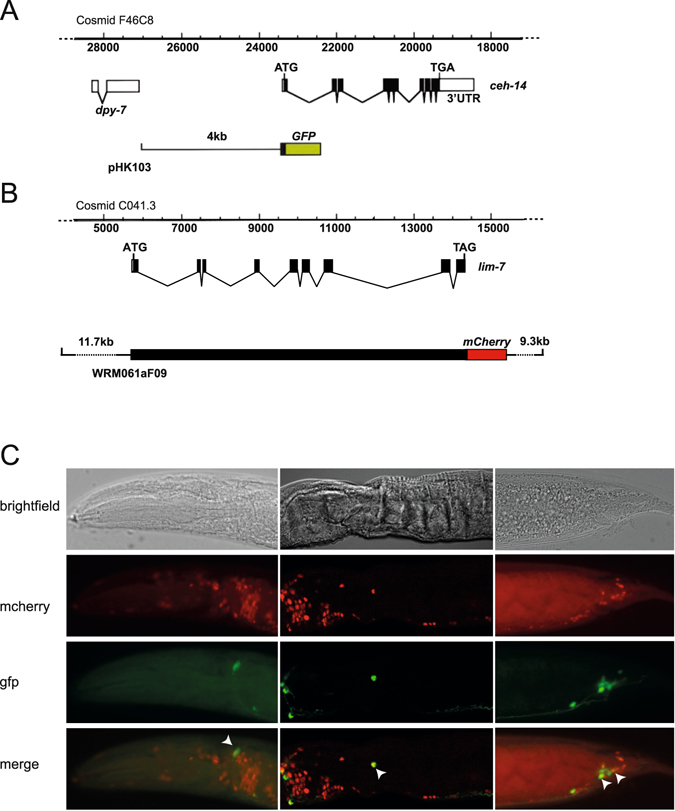
CEH-14 and LIM-7 are co-expressed in several *C. elegans* neurons. (**A**) The *ceh-14* locus within cosmid F46C8 is shown with exons indicated by black boxes and introns by lines. The *ceh-14::GFP* reporter construct in plasmid pHK103 is shown underneath. The diagram was modified from^27^. (**B**) The *lim-7* locus within cosmid C04F1 is shown with exons indicated by black boxes and introns by lines. The structure of the *lim-7::mCherry* reporter construct created by recombineering fosmid WRM061aF09 is shown underneath. **(C)** Fluorescence micrographs depict expression of the *ceh-14::GFP* (*chIs513*) and *lim-7::mcherry* (*stIs10289*) reporters in the head (left panels) and tail (right panels) of adult hermaphrodites of strain HRN073. The corresponding brightfield image is shown above and a merged image is shown below. Representative cells in which both gfp and mcherry fluorescence was observed are indicated by arrowheads.

To assess whether the nematode LIM-HD proteins CEH-14 and LIM-7 might function together like their vertebrate counterparts, we investigated the expression patterns of the *ceh-14* and *lim-7* genes in *C. elegans* using fluorescent reporter constructs. The *ceh-14* reporter construct contains 4 kbp immediately upstream of *ceh-14* and the first exon of *ceh-14*, which encodes the first 16 amino acids of CEH-14, fused to the gfp coding sequence (Fig. 6A). As there is no nuclear localisation sequence in this construct, expressing cells are fluorescent throughout the nucleus and cytoplasm. The *ceh-14::gfp* reporter has previously been reported to be expressed in the nematode spermatheca and in the nervous system. In the latter, expressing neurons in the head have been identified as the sensory neurons AFDL/R, the interneurons BDUL/R, and the interneuron ALA^45^. Neurons in the tail that express *ceh-14* have been identified as PVT, PVQL/R, DVC, PVNL/R, PVWL/R, PVR, PHAL/R, PHBL/R and PHCL/R^45^.

The *lim-7* reporter construct was made by recombineering of fosmid WRM061aF09 to fuse an mCherry tag and 3xFLAG epitope to the C-terminus of the LIM-7 protein (Fig. 6B)^21^. This construct thus encompasses the complete *lim-7* genomic locus and is expected to contain all relevant regulatory elements. In nematodes carrying this reporter construct, mcherry fluorescence has been reported in the gonadal sheath cells, in the URA neurons in the head, and in 10 additional cells near the isthmus and terminal bulb of the pharynx. Although the precise identities of the latter cells have not been defined, they are presumed to be neurons^21^.

We performed confocal microscopy on a strain carrying both the *ceh-14* and the *lim-7* reporter constructs described above (HRN073 *stIs10289*; *chIs513*) to further examine the expression patterns of *ceh-14* and *lim-7* and to assess whether there are any cells in which CEH-14 and LIM-7 are co-expressed. In this strain, *ceh-14* was only consistently observed in one cell in the head, which appears to correspond to the ALA cell. In the tail, expression of *ceh-14* matched previous reports. Expression of *lim-7* was observed in the gonadal sheath cells and in a number of cells surrounding the pharynx as previously reported. In addition, we observed expression in at least 12 cells in the tail.

GFP and mCherry images were acquired and overlayed using ImageJ software to identify cells expressing both *lim-7* and *ceh-14*. Co-expression was observed in one cell in the head, ALA (Fig. 6C left panels) and one cell positioned posterior to the pharynx, BDU (Fig. 6C middle panels). Co-expression was also observed in five cells in the tail, which are appropriately positioned to be PHAL/R, PHBL/R and PVT (Fig. 6C right panels).

## Discussion

Through yeast two-hybrid analyses, complemented by biophysical and structural characterisation, we have shown that the tandem LIM domains of CEH-14 and the LIM interaction domain of LIM-7 form a complex analogous to that of their mammalian counterparts, LHX3 and ISL1. Moreover, our *in vivo* data indicate that CEH-14 and LIM-7 are co-expressed in a subset of neurons in *C. elegans* suggesting that transcriptional events in these neurons could be regulated by interactions between the nematode Islet and LHX3-family LIM-HD transcription factors.

Interactions among the mammalian LIM-HD transcription factors as well as interactions between the LIM-HD transcription factors and other binding partners such as the LIM only proteins (LMO) and the LIM domain binding protein (LDB1) have been extensively investigated (reviewed in ref. 26). Mutagenic and structural studies of LHX3/4_LIM1+2_-ISL1/2_LID_ complexes as well as LMO_LIM1+2_-LDB1_LID_ and other LMO-LID complexes have shown that binding is modular. That is, binding is mediated by two distinct linear motifs (~8–10 residues each) in the LIDs (Fig. 2A), which contact equivalent faces on each of the partner LIM domains. The linear motifs in the LIDs are specific for their cognate LIM domains (i.e., the LIM2 domain does not bind the LIM1-binding motif and vice versa) and the variable length spacer that lies between the linear motifs tends to be plastic or disordered and makes little or no contribution to binding^11, 12, 31, 43, 46^.

In the case of the interaction between the LIM domains of CEH-14 and the LID of LIM-7, we observed that both LIM domains of CEH-14 are required for high affinity binding to LIM-7 (Fig. 2C). This requirement is a common feature of tandem LIM/LID interactions in which it has also been demonstrated that contributions to binding tend to be dominated by one or the other LIM domain, as evidenced by independent binding and/or abrogation of binding by mutagenesis^10, 11, 31, 47–49^. For example, mouse LHX3/4-ISL1/2 interactions are dominated by the association of the LIM2 domain of LHX3 with its cognate binding sequence^11, 12^; mutagenesis indicates that contacts with the LIM1 domain play an additional minor role in LHX3/4 interaction with ISL2^12^ (Fig. 5A). In line with these typical features of LIM/LID interactions, the LIM2 domain of CEH-14 showed weak independent binding to the LID of LIM-7 (Fig. 2C).

Consistent with the LIM2 domain of CEH-14 being the dominant binder, our mutagenesis experiments identified residues P350-M532 of the LIM-7 LID, which interface with LIM2 of CEH-14 in the homology model, as contributors to high affinity binding. In contrast with other characterised LIM/LID interactions, in which the spacer between the two linear motifs of the LID make minimal contributions to binding^11, 12, 31, 43, 46^, these same mutagenesis experiments also revealed an even more important role for the spacer region of the LIM-7 LID (H359-D361; Table 2; Fig. 2A). However, inspection of the homology model indicates that these hotspot residues predominately make contact with the LIM2 domain (which is consistent with that domain being the dominant binder), or form intramolecular interactions that could stabilise the structure of the LIM-7_LID_ (Fig. 5B). These data suggest that precise mechanisms for stabilisation of the interaction may have diverged for the mouse versus nematode LHX-Islet interactions. Other LIM-LID interactions are consistent with minor variation in modes of binding. For example, an extended or bipartite binding hotspot at the LIM2 interface is seen for LMO_LIM2_-partner interactions^26^, and the main binding hotspot for TES_LIM2+3_-ARP7A_LID_ completely overlaps the very short spacer in ARP7A_LID_^50^.

Beyond our biophysical data confirming the interaction of CEH-14 and LIM-7 *in vitro*, our expression analysis indicates that these two proteins are co-expressed in several neurons, presenting the possibility that CEH-14 and LIM-7 may co-ordinately regulate gene expression in nematode neuronal development. Although the neuronal expression of LIM-7 was previously reported, specific roles for LIM-7 in neuronal development or function have not yet been described. An important role for LIM-7 in the developing nervous system is suggested by the observation that the majority of *lim-7* mutant animals, which arrest at the first larval (L1) stage of development, show an uncoordinated phenotype^21^. In the case of CEH-14, specific developmental functions in several neurons have been described, including the AFD thermosensory neuron, the ALA sleep neuron and the BDU interneuron^20, 51, 52^. The latter two are of particular interest here since we have identified co-expression of CEH-14 and LIM-7 in these two cell types. In the ALA, CEH-14 regulates expression of several ALA-specific genes and the absence of CEH-14 renders worms unable to respond to sleep cues in the form of the epidermal growth factor LIN-3. In the BDU interneuron, CEH-14 regulates expression of a battery of neuropeptides. It remains to be determined whether LIM-7 works together with CEH-14 in these gene regulatory events. Nonetheless, our finding that these two proteins are co-expressed in these two cells and others suggests the possibility of coordinate activity.

As outlined earlier, the vertebrate homologues of LIM-7 and CEH-14 interact with distinct binding partners to regulate specific developmental programs. In V2 interneurons, a binary complex of LHX3 and LDB1 regulate expression of *chx10* while in motor neurons a ternary complex of LDB1, LHX3 and ISL1 targets *Hb9* ^7, 9^. The two cell populations possess additional mechanisms that suppress the alternative differentiation program through both protein-DNA and protein-protein interactions. For example, in chick and mice, *chx10* and *Hb9* encode homeodomain proteins that are thought to block the binding site of the ternary and binary complexes, respectively, thereby repressing inappropriate transcriptional activity^9^. In addition, in developing motor neurons the LIM only protein LMO4 appears to compete with LHX3 for binding of LDB1 to prevent formation of the binary complex^9, 53^. A similar regulatory mechanism was observed in Drosophila, in which dLMO antagonises the formation of a transcriptional complex comprising Apterous (the fly homologue of mammalian LHX2) and Chip (the fly homologue of LDB1) by competing for binding to Chip protein^14, 54^.

Analogous LIM-HD/LDB binary and ternary complexes involving CEH-14 and LIM-7 may similarly regulate neuronal development in *C. elegans*. Like mammalian LDB1, which is widely expressed in both embryonic and adult tissues^17^, the *C. elegans* homologue called LDB-1 is expressed broadly during nematode embryogenesis, with expression persisting into adulthood in some tissues including the gonadal sheath cells and body wall muscle cells^27^. Of particular relevance here is the observation that LDB-1 is expressed throughout the nervous system in both larvae and adults, and is therefore present with CEH-14 and LIM-7 in a subset of neurons. The LIM interacting domain of LDB-1 shares ~60–65% sequence identity with its mammalian homologues, suggesting functional conservation^27^. Consistent with this, LDB-1_LID_ was shown to interact with CEH-14_LIM1+2_ in Y2H assays^27^.

Binary and ternary complexes involving CEH-14, LIM-7 and LDB-1, similar to those in mammals and Drosophila, may thus regulate analogous biological processes in *C. elegans*. However, the regulation of complex assembly that has been exhibited previously by LMO proteins is unlikely to be relevant in the nematode as *C. elegans* lacks LMO orthologs.

Downstream of the LHX3/LDB1 and LHX3/ISL1/LDB1 transcriptional complexes are the target genes *Hb9* and *Chx10*. The *C. elegans* homologue of Hb9, CEH-12, is expressed in the V_B_ subclass of motor neurons^55^ and the Chx10 homologue, CEH-10, is expressed in distinct group of neurons that includes several interneurons and motor neurons^56^. Whether expression of CEH-12 and CEH-10 is regulated by the analogous nematode LIM-HD/LDB binary and ternary complexes has not yet been examined.

In conclusion, this study has shown that a LIM-HD transcriptional complex that regulates differentiation of post-mitotic motor neurons in vertebrates is likely to also exist in *C. elegans*. The physical characteristics of protein-protein interactions within a CEH-14/LIM-7 assembly are essentially identical to that of vertebrate LHX3/ISL1 complexes. Although the function of that complex in driving cell fate decisions is likely similar to its mammalian counterpart, it is yet to be confirmed if mammals and nematode share regulatory mechanisms of cell-specific transcriptional complex assembly. The nematode and mammalian families of LIM-HD factors are very distantly related making it likely that Islet/LHX3 transcriptional assemblies are strongly conserved throughout bilateral metazoans and developed at an early stage of evolution of multicellular organisms.

## Materials and Methods

### Cloning and mutagenesis

All constructs were generated via standard or overlap extension PCR methods and cloned into pGBT9 and pGAD10 for yeast two-hybrid experiments, or pGEX-2T for biophysical *in vitro* work. All plasmids were sequenced to confirm identity (SUPAMAC, Royal Prince Alfred Hospital, Sydney). Constructs for protein expression were generated as fusions of CEH-14_LIM1+2_(CEH-14 residues 42–168; UniProt accession P20271) and LIM-7_LID_ (LIM-7 residues 347–376; UniProt accession G5EC36) where the two domains are connected by an 11-residue glycine-serine linker as previously described for LHX3_LIM1+2_-ISL1_LID_ fusion constructs^29, 57^.

### Recombinant protein expression and purification

Proteins were expressed with a glutathione S-transferase (GST) tag using a pGEX-2T vector in *Escherichia coli* BL21 (DE3) cells. Bacterial cell cultures in Luria broth supplemented with 100 μg/mL ampicillin were induced at mid-log phase (OD_600nm_ = 0.5–0.6) by the addition of 0.4 mM IPTG and incubated at 20 °C for 16–20 h. The proteins were purified by glutathione (GSH) affinity chromatography using the Sepharose4B resin (GE Healthcare) in 50 mM Tris (pH 8.0), 200 mM NaCl, 7 mM β-mercaptoethanol. The GST-tag was removed by mixing the beads overnight at 4 °C in the same buffer supplemented with 2.5 mM CaCl_2_ and 50U thrombin (Sigma-Aldrich). The eluted protein was further purified by size exclusion chromatography using a HiLoad^TM^ Superdex^TM^ S75 16/60 size exclusion column (GE Healthcare) equilibrated in 20 mM Tris, 150 mM NaCl, 1 mM DTT (pH 8.0) or 50 mM Tris, 100 mM NaCl, 2 mM TCEP (pH 8.0).

### Yeast two-hybrid analysis

pGBT9 and pGAD10 plasmids were co-transformed into AH109 cells (Clontech), as described previously^31^. All selective media lacked leucine and tryptophan (-L-W) to ensure co-transformation of bait and prey plasmids was maintained. For screening of interactions, media were further deficient in histidine (-H-L-W) but contained or lacked additional reagents for detection of different affinity interactions. Selective media supplemented with 40 μg/mL X-α-gal (Progen), further supplemented with 1 mM 3-amino-1,2,4-triazole (3-AT; Sigma), or, additionally deficient in adenine, were used to probe for weak, moderate and high affinity interactions. Transformed yeast colonies were cultured in the appropriate media, adjusted to A_600 nm_ = 0.2, and two serial 1:10 dilution suspensions prepared designated as 10^0^, 10^−1^, and 10^−2^, respectively. 2-μL aliquots of all three dilutions were spotted onto plates and incubated at 30 °C for 72 h.

### Circular dichroism analysis

Far UV-CD used a sample concentration of 5 μM protein in 10 mM Tris pH 8.5, 150 mM NaFl, 0.5 mM TCEP in a 1-mm path length quartz cell seated in a water-jacketed cell holder. Spectra were recorded at 20 °C on a Jasco J-720 spectropolarimeter equipped with a Neslab RTE-111 temperature controller. CD data were collected over the wavelength range 195–240 nm, with a speed of 20 nm/min, step resolution of 1 nm, bandwidth of 1 nm and a response time of 1 s. The final spectrum was the average of five scans, and was baseline corrected. Spectra collected previously^12^ were further normalised at 207 nm to account for small variations in protein concentration for comparison.

### Multiple angle laser light scattering

Size exclusion chromatography multiple angle laser light scattering (SEC-MALLS) analysis was performed using a SuperoseTM Peptide column attached to the AKTA HPLC system at a flow rate of 0.5 mL/min in 20 mM Tris.HCl, pH 8.0, 150 mM NaCl, 1 mM DTT). A protein sample of 200 μM was used. The size exclusion chromatography column was followed in-line by a miniDAWN light scattering detector and an interometric refractometer (Wyatt Technologies, Santa Barbara, CA). Light scattering analysis was performed using a 690 nm wavelength laser. Voltage and light scattering intensity were calibrated with toluene yielding a constant of 8.534 × 10^−6^ for this study. A refractive index increment (dn/dc) estimate of 0.19 mL/g was used for protein concentration determination^58^ and data were analysed using ASTRA software (Wyatt Technologies).

### Small angle X-ray scattering

Small-angle X-ray scattering data *I*(*s*) vs *s*, where *s* = 4πsin*Θ*/λ nm^−1^; 2*Θ* is the scattering angle and λ is the X-ray wavelength were collected from a sample of CEH-14–LIM-7 at 5.2 mg/mL in 50 mM Tris, 100 mM NaCl, 5 mM TCEP (pH 8.0) and a corresponding matched solvent blank that was prepared via dialysis^34^. The final post-dialysis sample concentration was determined using an A_280nm_ extinction coefficient of 14878 M^−1^ cm^−1^ calculated from the amino acid sequence of the protein using ProtParam^59^. SAXS data were recorded on a SAXSess (Anton Paar) Kratky camera (line collimation, 10 mm slit) equipped with a sealed tube source (Cu-*Kα*, λ = 1.5418 Å) and a CCD detector^60, 61^ through an *s*-range of 0.07–6 nm^−1^. The protein sample or matched solvent blank (30 μL) were mounted in the same quartz capillary (1 mm diameter), and irradiated at 10 °C for at total exposure time of 1 h (4 × 15 min blocks). The 2D scattering data were reduced to 1D *I*(*s*) vs *s* profiles using the SAXSQuant 2.0 software package (Anton Paar, Austria) taking into account sample absorbance and detector sensitivity. The scattering from the matched solvent was subtracted from the sample scattering to generate the smeared *I*(*s*) vs *s* profile of the protein in solution. The information content of the resulting scattering profile and effective *s*-range were assessed using *SHANUM* ^62^ and data were accordingly truncated to working *s*_max_ of 3.5 nm^−1^. All data were placed on an absolute scale (*I*(*s*), cm^−1^) using the scattering from water as a reference^63^. The partial specific volume and X-ray contrast were calculated using MULCh^61^.

The indirect Fourier transform of the SAXS data and subsequent calculation of the real-space *p*(*r*) vs *r* profile was performed using *GNOM* ^64^ in *PRIMUSQT* as part of the ATSAS 2.8 software package (https://www.embl-hamburg.de/biosaxs/software.html)^65^. The effects of the 10 mm slit beam geometry were taken into account using the prepared experimental beam profile as input to generate desmeared (i.e., beam-geometry corrected) *p*(*r*) vs *r* and SAXS profiles. The *R*_*g*_ and forward scattering intensity at zero angle, *I*(0), were evaluated from both *p*(*r*) and Guinier analysis (in the Guinier limit 0.16 < *sR*_*g*_ < 1.25)^35^. Porod volume estimation from the data was performed using *DATPOROD* ^65^ while the expected dry volume was calculated from the amino acid sequence using *SEQSTAT* (ATSAS 2.8). Concentration independent *M*_r_ estimates were evaluated using the methods of Fischer *et al*. (*SAXSMOW*)^32^ and Rambo and Tainer (volume of correlation, Vc)^33^. Automated shape classification of the *p*(r) vs *r* profile was calculated using *DATCLASS* (ATSAS 2.8). The *a priori* assessment of the non-uniqueness of the SAXS data was calculated suing *AMBIMETER* ^37^ which also produced a likely model-independent shape topology of the protein. Subsequent dummy atom bead model refinements were performed using *DAMMIF* ^38^. As shape restoration from SAXS data may be ambiguous (CEH-14–LIM-7 *AMBIMETER* score = 2.5; highly ambiguous), *DAMMIF* was run 10 times and the resulting individual models were assessed for consistency using the *DAMAVER* set of programs^40^ that calculate all pair-wise spatial superpositions, the normalised spatial discrepancy of the alignments (where NSD < 0.7 are spatially similar) and a final averaged 3D-representation of the protein (corrected for volume and bead-occupancy). The resolution of the individual bead-model cohort was assessed using *SASRES* ^41^. *CRYSOL* ^66^ was employed to calculate the SAXS profiles and evaluate the fits to the desmeared SAXS data of the LHX3-ISL1 X-ray crystal structure or the CEH-14–LIM-7 homology model and the discrepancy was assessed using the reduced *χ* ^2^ test. Additional data-model comparisons were performed using the Correlation Map method^39^ (set to a significance threshold α of 0.01) which is independent of correct error estimation and propagation.

### Nuclear Magnetic Resonance Spectroscopy

Spectra were acquired at 298 K on a 600 MHz Bruker Avance III spectrometer equipped with a 5-mm TCI CryoProbe (Bruker). ^15^N-labelled^67^ CEH-14–LIM-7 was generated and buffer-exchanged into 20 mM HEPES pH 7.0, 1 mM DTT supplemented with 10% D_2_O and 20 μM DSS (4,4-dimethyl-4-silapentane-1-sulfonic acid). ^15^N- HSQC experiments were performed using the standard pulse sequence hsqcf3gpsi from the Bruker library. Spectra were processed with TopSpin (Bruker) and analysed with Sparky (T.D. Goddard and D.G. Kneller, SPARKY 3, University of California, San Francisco).

### Homology Modelling

The CEH-14–LIM-7 model was generated in SwissModel^68^ using the sequence of the CEH-14–LIM-7 construct as the target and 2RGT Chain B as the template. The sequence identity based on the structured regions of ISL1-LHX3 was 46%.

### Analysis of fluorescent reporter genes in *C. elegans*

*C. elegans* strains were cultured and maintained using standard protocols^69^. Strain RW10289 *stIs10289*[*lim-7*(+)::GL-mCherry-3XFLAG, *unc-119*(+)] was provided by Dr Laura G. Vallier (Department of Biology, Hofstra University, NY, USA)^21^. Strain TB513 *chIs513*[pHK103(*ceh-14*::gfp), pMH86 (*dpy-20*(+))]; *dpy-20*(*e2071*) was provided by Hiroshi Kagoshima (National Institute of Genetics, Mishima, Japan)^45^. Strain HRN073 *stIs10289*; *chIs513* was generated by crossing strains RW10289 and TB513. Reporter gene expression in nematode strain HRN073 was visualised using an Olympus FluoView^TM^ FV1000 confocal microscope (GFP, 488 nm; mcherry, 568 nm) (Australian Centre for Microscopy and Microanalysis, University of Sydney).

### Data Availability

The homology model, bead model and SAXS data for CEH-14–LIM-7 have been deposited to the Small Angle Scattering Biological Database (SASBDB)^42^ under the accession code SASDC22. All other data generated or analysed during this study are included in this published article (and its Supplementary Information files or are available from the corresponding authors on reasonable request).

## Electronic supplementary material

Supplemental Data
