# Supplementary material for: Interactions between LHX3- and ISL1-family LIM-homeodomain transcription factors are conserved in Caenorhabditis elegans
Source: Sci Rep. 2017 Jul 4;7:4579. doi: 10.1038/s41598-017-04587-8 (PMC5496915; doi:10.1038/s41598-017-04587-8)
Supplement: Supplementary file 1 — Supplemental Data [file 41598_2017_4587_MOESM1_ESM.pdf]

Interactions between LHX3- and ISL1-family LIM-homeodomain transcription factors are conserved in *Caenorhabditis elegans*

[1,3]Mugdha Bhati, [1,4]Estelle Llamosas, [1,6]David Jacques, [1,7]Cy M Jeffries, [2]Siavoush Dastmalchi, [1,5]Nina Ripin, [1*]Hannah R Nicholas, [1*]Jacqueline M Matthews.

SUPPLEMENTAL DATA

Supplementary Data 1

ISL1 and LHX3 sequences used for comparison

Highlighted sequences were used as the target sequences in PBLAST comparisons to generate the % sequence identities presented in Figure 1D.

ISL sequences

LIM1+2

HD

LID

Hs >sp|P61371|ISL1_HUMAN Insulin gene enhancer protein ISL-1 OS=Homo sapiens GN=ISL1 PE=1 SV=1
MGDMGDPPKKKRLISLCVGCGNQIHDQYILRVSPDLEWHAACLKCAECNQYLDESCTCFVRDGKTYCKRDYIRLY
GIKCAKCSIGFSKNDFVMRARSKVYHIECFRCVACSRQLIPGDEFALREDGLFCRADHDVVERASLGAGDPLSPL
HPARPLQMAAEPISARQPALRPHVHKQPEKTTRVRTVLNEKQLHTLRTCYAANPRPDALMKEQLVEMTGLSPRVI
RVWFQNKRCKDKKRSIMMKQLQQQQPNDKTNIQGMTGTPMVAASPERHDGGLQANPVEVQSYQPPWKVLSDFALQ
SDIDQPAFQQLVNFSEGGPGSNSTGSEVASMSSQLPDTPNSMVASPIEA

Hs Isl2 >sp|Q96A47|ISL2_HUMAN Insulin gene enhancer protein ISL-2 OS=Homo sapiens GN=ISL2 PE=1 SV=1
MVDIIFHYPFLGAMGDHSKKKPGTAMCVGCGSQIHDQFILRVSPDLEWHAACLKCAECSQYLDETCTCFVRDGKT
YCKRDYVRLFGIKCAKCQVGFSSSDLVMRARDSVYHIECFRCSVCSRQLLPGDEFSLREHELLCRADHGLLLERA
AAGSPRSPGPLPGARGLHLPDAGSGRQPALRPHVHKQTEKTTRVRTVLNEKQLHTLRTCYAANPRPDALMKEQLV
EMTGLSPRVIRVWFQNKRCKDKKKSILMKQLQQQHSDKTSLQGLTGTPLVAGSPIRHENAVQGSAVEVQTYQPP
WKALSEFALQSDLDQPAFQQLVSFSESGSLGNSSGSDVTSLSSQLPDTPNSMVPSPVET

Mm >sp|P61372|ISL1_MOUSE Insulin gene enhancer protein ISL-1 OS=Mus musculus GN=Isl1 PE=1 SV=1
MGDMGDPPKKKRLISLCVGCGNQIHDQYILRVSPDLEWHAACLKCAECNQYLDESCTCFVRDGKTYCKRDYIRLY
GIKCAKCSIGFSKNDFVMRARSKVYHIECFRCVACSRQLIPGDEFALREDGLFCRADHDVVERASLGAGDPLSPL
HPARPLQMAAEPISARQPALRPHVHKQPEKTTRVRTVLNEKQLHTLRTCYAANPRPDALMKEQLVEMTGLSPRVI
RVWFQNKRCKDKKRSIMMKQLQQQQPNDKTNIQGMTGTPMVAASPERHDGGLQANPVEVQSYQPPWKVLSDFALQ
SDIDQPAFQQLVNFSEGGPGSNSTGSEVASMSSQLPDTPNSMVASPIEA

Gg >sp|P50211|ISL1_CHICK Insulin gene enhancer protein ISL-1 OS=Gallus gallus GN=ISL1 PE=1 SV=1
MGDMGDPPKKKRLISLCVGCGNQIHDQYILRVSPDLEWHAACLKCAECNQYLDETCTCFVRDGKTYCKRDYIRLY
GIKCAKCSIGFSKNDFVMRARAKVYHIECFRCVACSRQLIPGDEFALREDGLFCRADHDVVERASLGAGDPLSPL
HPARPLQMAAEPISARQPALRPHVHKQPEKTTRVRTVLNEKQLHTLRTCYAANPRPDALMKEQLVEMTGLSPRVI
RVWFQNKRCKDKKRSIMMKQLQQQQPNDKTNIQGMTGTPMVAASPERHDGGLQANPVEVQSYQPPWKVLSDFALQ
SDIDQPAFQQLVNFSEGGPGSNSTGSEVASMSSQLPDTPNSMVASPIEA

Xl >tr|Q08B34|Q08B34_XENLA Islet-1 OS=Xenopus laevis GN=isl1 PE=2 SV=1
MGDMGDPPKKKRLISLCVGCGNQIHDQYILRVSPDLEWHAACLKCAECSQYLDESCTCFVRDGKTYCKRDYIRLY
GIKCAKCNIGFSKNDFVMRARSKVYHIECFRCVACSRQLIPGDEFALREDGLFCRADHDVVERASLGGSDPLSPL
HPGRPLQMAAEPICARQPALRPHVHKQPEKTTRVRTVLNEKQLHTLRTCYAANPRPDALMKEQLVEMTGLSPRVI
RVWFQNKRCKDKKRSILIKQLQQQQPNDKTNIQGMTGTPMVASSPERHDGGLQANPVEVQTYQPPWKVLSDFALQ
SDIDQPAFQQLTCFSQVNFSEGGPGSNSTGSEVASMSSQLPDTPNSMVASPIEA

Dr >sp|P53405|ISL1_DANRE Insulin gene enhancer protein isl-1 OS=Danio rerio GN=isl1 PE=2 SV=1
MGDMGDPPKKKRLISLCVGCGNQIHDQYILRVSPDLEWHAACLKCAECNQYLDESCTCFVRDGKTYCKRDYIRLY
GIKCAKCNIGFSKNDFVMRARSKVYHIECFRCVACSRQLIPGDEFALREDGLFCRADHDVVERATMGAGDPLSPL
HPARPLQMAAEPISARQPALRPHVHKQPEKTTRVRTVLNEKQLHTLRTCYNANPRPDALMKEQLVEMTGLSPRVI
RVWFQNKRCKDKKRSILMKQLQQQQPNDKTNIQGMTGTPMVATSPERHDGGLQANQVEVQSYQPPWKVLSDFALQ
SDIDQPAFQQLVNFSEGGPGSNSTGSEVASMSSQLPDTPNSMVASPIEA

Ci >tr|Q4H3A5|Q4H3A5_CIOIN Transcription factor protein OS=Ciona intestinalis GN=Ci-isl PE=2 SV=1

MNESFAIFGQDEKLGAKMADPHGGCVSASLNCDPCRIPLCVGCGSPIHDQYILRVAPNLEWHAGCLKCADCGQYL
DETCTCFVRDGKTYCKRDYTRLFGTKCNKCGLCFSKNDFVMRARDKIYHIQCFKCVACSRQLIPGDEFALRDDGL
FCKADHEVATSGDMMVHDGHMIPGIPQTPNPQGVISPQMGGERVISHRSGGHSGGQRRSKDAKTTRVRTVLNEKQ
LHTLRTCYAANCRPDALMKEQLTEMTGLSSRVIRVWFQNKRCKDKKRSIALKQIQEQQAKQQHNNEQGNNVQGLS
GMNGVPMVASEPVRNDNSVSVAPVEVRNYQQPAWKALSDFALQSEIEQPAFQQLMNNFSDQGQGSISDSSEISSI
PSVSSASMDSTTCSTPHTVESSVPACS

PdIsl >tr|A6YB99|A6YB99_PLADU Islet OS=Platynereis dumerilii PE=2 SV=1
MATEPQAKGQILYTERKHSSMCVGCGSKIQDQYILRVAPDLEWHAACLKCADCDQFLDETCTCFVREGKTYCKRD
YARLFGTKCARCTESFSKNDFVMRARNKIYHIDCFRCVACSRQLIPGDEFALRDDGLFCKSDHEVLERAQNGATP
TPENNNNNVNALNNNEKEIKTEKTGSIKSGQAEHGSRRGNSGHKSEHKPTRVRTVLNEKQLHTLRTCYNANPRPD
ALMKEQLTEMTGLSPRVIGVWFQNKRCKDKKRSIALKQMQEQQHKNGTLGRPLSGVPMVASSPVRHESNLQPNPV
EVTSYQPPWKALSEFATQSELDQPPFHQLEHAMFLRGEEDKLMYHGYRSDLDSHHHTMNSFDSPESMNGGYTGMS
DNQTPPGSMYLDDYSQPPNSVSSSELPPTPSELSSPMSQ

DmIsl >tr|Q9VJ37|Q9VJ37_DROME Tailup, isoform A OS=Drosophila melanogaster
GN=tup PE=4 SV=1
MVMAEIGGHLAHQLPLHNHNHNQTGLQPSLVMNHHLDLDCHGHDVIKKQRLSHCVGCGGQIHDQYILRVAPDLEW
HAACLKCQECRQFLDESCTCFVRDGKTYCKRDYVRLFGTKCDKCGNSFSKNDFVMRAKTKIFHIECFRCSACARQ
LLPGDEFALRDAGALYCKEDHDVLEKSSQSSLTSSSVESNNNISSSNNNNTNLSNNNHSSELGSMSDSGSESGSH
KSIRDKRPSGPSDGKPTRVRTVLNEKQLHTLRTCYNANPRPDALMKEQLVEMTSLSPRVIRVWFQNKRCKDKKKT
IQMKLQMQQEKEGRKLGYGAMQGIPMIASSPVRHDSPLNLQGLDVQTYQPPWKALSDFALHADLDSNGAINTHTP
AFQQLVNQMHGYDLNGMPILPPHPHSHPAQGPPHQHPPPPGGPHNHQNQQPNQQPGGSSLDSGITSHHHPDSTDS
YVTYLESDDKSKLALTPSSSSSASAGTSISSPPSGVGAGGGGAVGGGSGVLGLGVVANQSATEQLMQMLQKVTGS
ASPASHAVL

CeIsl >tr|G5EC36|G5EC36_CAEEL CeLIM-7 (Fragment) OS=Caenorhabditis elegans
GN=lim-7 PE=2 SV=1
MNICMRNGYEQFSLTSPGTSDLEIGGSFWKDEPDTKYLCLDSPVEQRQHQPPMAVCAGCRLEISDRYFLRVNPNL
EFHAQCLKCVQCSRPLDENQTAFVKNGQTYCRDDYRRLFTTRCSRCHGDFDKTDLVMRAGPQNVFHLNCFACVAC
EKRLQTGEEFQIKNNSLYCRSDCRGLDNPDTSASVPDYSKLNNNNNNDNNNSSSNFDEDEWDEERSTLTSLDNNT
SSPLGSPKSDGVRTPLFGHHNSGSGGSTSSCGKKKKDKQATRVRTVLNENQLKILRDCYSINSRPDATLKERLVE
MTGLSARVIRVWFQNKRCKDKKRQIQITENRLNSEREEVLNRVRVNGIGPLMVQPATPHIDNTLGGPIDIQHFAQ
WNGTPPPPPPPQYGNPMMFNSPSTFDVSVILAPVAPNVTSPSEALGPLGASVFPHFSPQHAPFTATSHDISSPAPC
GE

NvIsl >NvIslet (161808) protein prediction
MPICCSFSAEKRRVSMCVGCGSQIHDQYILRVAPDLEWHASCLKCADCHMYLDEKCTCFVREGKTYCKRDYVRLF
GTKCAKCSLNFSKNDFVMRARNKIYHIDCFRCVACSRQLVPGDEFALREDGLFCKADHEIVEKATATAQATHVRN
NGQRSSQSGGQTNGQTTPDSTPSKRKTDRPTRVRTVLNEKQLHTLRTCYNANPRPDAMMKEQLVEMTGLSPRVIR
VWFQNKRCKDKKKNPNSLSPTGAPPRTLPQQTLPAISGVPYGNSGSMRPDAMSDMSGYQPWKALNDFAMHSEMDQ
GAFQQLVHFSEQAPGSLPPSSDGIVSTGN*

MlIsl >GenBank: JF912806.1_MNELE TALE class homeobox transcription factor
Isl OS=Mnemiopsis leidyi GN=Isl PE=2 SV=1
MNDKGVNPSVCAGCRCPITDQFILRVAPNLEWHASCLKCDDCNKFLDENCTCFIREGKPYCKKDFVRFGAKCHRC
DQGFSSNDFVMRVRENIFHLSCFRCNMCSRQLVPGEEFALLPEGLICGTHIKQQHHQQAPLPNEPLPESKPRSTN
TGSSGEQKTTRVRTVLNDRQLRILRTCYNNNPRPDALMKEQMTKLTGLSARVIRVWFQNKRCKDKKKAIAAASGD
DVISSSVADSPSEDRLKSGMESLSEKDFSSLDCSQIDFNNSSTSTSPALSATGDTYSMYDANKDARSLWNTGDFI
PSYPQDDSVSGHLGSSFEDNK

AqIsl > PREDICTED: insulin gene enhancer protein ISL-1-like [Amphimedon
queenslandica]NCBI Reference Sequence: XP_019855411.1
MMECENSNINYNCNNAVDAVMDSFTTQRICHGCKLSIEDEFSLHVSPNLDWHVSCLICCECHDFMDENCETCFIK
DGRPYCRTDYIRLFGTQCKRCSEPINADTLVMRAKKEIFHVDCFCCTLCDKKLSTGEQFGMAGGKLYCKADFESL
PIDSIEPTMEAELVESPETLLHNGCTQSIATTSSPSTSSQATSTTTATSKSSDKRRSSTEQKQPRIRTVLTEQQL
QTLRSVYQTNPRPDALLKEQLCELTGLSPRVIRVWFQNRRCKDKKALQKAEAARLQSGGKVLPMTPQTPMTPDNM
SQVSPHGGMQAFTYPLPAYDHITTPTLPPTPSNTCVSPIVSYQHNPPLWASPTDTVSPFDHAVDFSSPVPITTEH
QSYIYSSASLQPHMQQPTTVTHFSF

TaIsl >TaIsl (30944) protein prediction[1]
MLILISFFECPDQLPFCSGCGGKINDRYILQVAPDMQYHAACLKCASCQQLLDEKETCFLRNGKPYCKSDFKMLF
HNRCTKCNRIFEPSEFIMRAKGNPYHIDCFRCHSCMRKLIPGDRYGVDTYILYCKEHYLNKMSSSSNHDTLQSTM
ADSDWQNSDNTDTKSQSTHAKSKQLTSRKGMKGTRIRTVLNEKQLQTLRSYYASNPRPDSTVKEKLVELTGLNPR
VIRVWFQNKRCKDKKIKAAGEQALEEEVVRIILQYTVSIC*

LHX3 Sequences

Hs >sp|Q9UBR4|LHX3_HUMAN LIM/homeobox protein Lhx3 OS=Homo sapiens GN=LHX3 PE=1 SV=2
MLLETGLERDRARPGAAAVCTLGGTREIPLCAGCDQHILDRFILKALDRHWHSKCLKCSDCHTPLAERCFSRGES
VYCKDDFFKRFGTKCAACQLGIPPTQVVRRAQDFVYHLHCFACVVCKRQLATGDEFYLMEDSRLVCKADYETAKQ
REAEATAKRPRTTITAKQLETLKSAYNTSPKPARHVREQLSSETGLDMRVVQVWFQNRRAKEKRLKKDAGRQRWG
QYFRNMKRSRGGSKSDKDSVQEGQDSDAEVSFPDEPSLAEMGPANGLYGSLGEPTQALGRPSGALGNFSLEHGGL
AGPEQYRELRPGSPYGVPPSPAAPQSLPGPQPLLSSLVYPDTSLGLVPSGAPGGPPPMRVLAGNGPSSDLSTGSS
GGYPDFPASPASWLDEVDHAQF

HsLhx4 >sp|Q969G2|LHX4_HUMAN LIM/homeobox protein Lhx4 OS=Homo sapiens GN=LHX4 PE=1 SV=2
MMQSATVPAEGAVKGLPEMLGVPMQQIPQCAGCNQHILDKFILKVLDRHWHSSCLKCADCQMQLADRCFSRAGSV
YCKEDFFKRFGTKCTACQQGIPPTQVVRKAQDFVYHLHCFACIICNRQLATGDEFYLMEDGRLVCKEDYETAKQN
DDSEAGAKRPRTTITAKQLETLKNAYKNSPKPARHVREQLSSETGLDMRVVQVWFQNRRAKEKRLKKDAGRHRWG
QFYKSVKRSRGSSKQEKESSAEDCGVSDSELSFREDQILSELGHTNRIYGNVGDVTGGQLMNGSFSMDGTGQSYQ
DLRDGSPYGIPQSPSSISSLPSHAPLLNGLDYTVDSNLGIIAHAGQGVSQTLRAMAGGPTSDISTGSSVGYPDFP
TSPGSWLDEMDHPPF

MmLHX3 >sp|P50481|LHX3_MOUSE LIM/homeobox protein Lhx3 OS=Mus musculus GN=Lhx3 PE=1 SV=1
MLLEAELDCHRERPGAPGASALCTFSRTPEIPMCAGCDQHILDRFILKALDRHWHSKCLKCSDCHVPLAERCFSR
GESVYCKDDFFKRFGTKCAACQLGIPPTQVVRRAQDFVYHLHCFACVVCKRQLATGDEFYLMEDSRLVCKADYET
AKQREAEATAKRPRTTITAKQLETLKSAYNTSPKPARHVREQLSSETGLDMRVVQVWFQNRRAKEKRLKKDAGRQ
RWGQYFRNMKRSRGSSKSDKDSIQEGQDSDAEVSFTDEPSMADMGPANGLYSSLGEPAPALGRPVGGLGSFTLDH
GGLTGPEQYRELRPGSPYGIPPSPAAPQSLPGPQPLLSSLVYPDTNLSLVPSGPPGGPPPMRVLAGNGPSSDLST
ESSSGYPDFPASPASWLDEVDHAQF

GgLHX3 >sp|P53412|LHX3_CHICK LIM/homeobox protein Lhx3 OS=Gallus gallus GN=LHX3 PE=2 SV=1
MLLERVRAGSEKAAELCPFPRSPEIPLCAGCNQHIVDRFILKVLDRHWHSKCLKCSDCQTQLAEKCFSRGDGVYC
KEDFFKRFGTKCAACQQGIPPTQVVRRAQDFVYHLHCFACIVCKRQLATGDEFYLMEDSRLVCKADYETAKQREA
ESTAKRPRTTITAKQLETLKNAYNNSPKPARHVREQLSSETGLDMRVVQVWFQNRRAKEKRLKKDAGRQRWGQYF
RNMKRSRGTSKSDKDSIQEEGPDSDAEVSFTDEPSMSEMSHSNGIYSNLSEASPALGRQAGTNGGFSLDHSGIPA
QDQYHDLRSNSPYGIPQSPASLQALPGHQPLISSLVYPDSGLGIMGQGGQGVPQSMRVLAGNGPSSDLSTGSSGG
YPDFPASPASWLDEVDHAQF

XlLHX3 >sp|P36200|LHX3_XENLA LIM/homeobox protein Lhx3 OS=Xenopus laevis GN=lhx3 PE=1 SV=1
MLLERVRTGTQKSSDMCGYTGSPEIPQCAGCNQHIVDRFILKVLDRHWHSKCLKCNDCQIQLAEKCFSRGDSVYC
KDDFFKRFGTKCAACQQGIPPTQVVRRAQEFVYHLHCFACIVCKRQLATGDEFYLMEDSRLVCKADYETAKQREA
ESTAKRPRTTITAKQLETLKNAYNNSPKPARHVREQLSSETGLDMRVVQVWFQNRRAKEKRLKKDAGRQRWGQYF
RNMKRSRGNSKSDKDSIQEEGPDSDAEVSFTDEPSMSEMNHSNGIYNSLNDSSPVLGRQAGSNGPFSLEHGGIPT
QDQYHNLRSNSPYGIPQSPASLQSMPGHQSLLSNLAFPDTGLGIIGQGGQGVAPTMRVIGVNGPSSDLSTGSSGG
YPDFPVSPASWLDEVDHTQF

DrLHX3 >sp|Q90421|LHX3_DANRE LIM/homeobox protein Lhx3 OS=Danio rerio GN=lhx3 PE=2 SV=1
MLLEHPGSSCQNAGNYTRYSSSQDIPVCAGCNQHIVDRFILKVLDRHWHSKCLKCSDCQSQLADKCFSRGDSVYC
KDDFFKRFGTKCAACQQGIPPTQVVRRAQDFVYHLHCFACIVCKRQLATGDEYYLMEDSRLVCKADYETAKQREA
DSTAKRPRTTITAKQLETLKNAYNNSPKPARHVREQLSTETGLDMRVVQVWFQNRRAKEKRLKKDAGRQRWGQYF
RNMKRSRGTSKSDKDSTQEDGMDSDAEVSFTDEPPMSDLGHSNGIYSSLSESSPALSRQGGNHPAFPLEHGAIIP

SQEPYHDIQASSPYSLPQSPGPLQPLPRHQPLISSLVYPESGLPMAGQSGGQDMTPGVRMMAAGNGPSSDLSTGS
SGGYPDFPASPASWLDEVDHAQF

CiLHX3 >tr|F6QIP6|F6QIP6_CIOIN Uncharacterized protein OS=Ciona intestinalis GN=lhx3 PE=4 SV=2
MQTGSEFHQNSAGSIRHQPDIAYHQDRKHLSTQQWDDNGKKSDVNQDDDFDQYVDDDVGYDVGDDFDDDDDDDGI
VVDCDDDRDSLMDLDFSTSLLSGHSGDFQDRKHDFPTSSLQELFSMTQSVTSSSCDVTNLVTSSIQSHPDLNDSG
IVTTVGESPQNPFLPQTSSKQTDSAGSLFALLSSDQRIQAKIPKCTGCDHHIFDRYILKVQDKPWHSQCLKCNDC
GRQLTDKCFSRGSYVYCKEDFFKRFGTKCSGCELAIPPTQVVRRAQDNVYHLECFRCFMCSEQLGTGDQFYLLDD
SRLVCKKDYEHAKSRDLDMDNGIKRPRTTITAKQLETLKIAYNQSPKPARHVREQLSSDTGLDMRVVQVWFQNRR
AKEKRLKKDTGRQRWGELFRSGAPSSGPHCRPNPDSPPSGGKRRVGGHSNRKRPSSSPGGTRIAIPIPSSIQSPG
RAPTNGQIESNFIAPEHNAPPHDGIMMGESPCFAPGEVPYPQQSSNHAYLSPGSIPDMGGFPNLTRNYDYVDGPQ
ILGPGMAPIMKPPTNNAIPNYYVTSPQMHHNQHQKNDCDVISESSGHSNLSDLSSSPRSWLGELDHVTHFQ

PdLHX3 – at the time of writing no LHX3 family protein was found in generally available public databases. However, the reference genome has not yet been published for Platynereis dumerilii.

DmLHX3 >tr|Q9VJ00|Q9VJ00_DROME FI01025p OS=Drosophila melanogaster GN=Lim3 PE=2 SV=2
MMQTLKPPPPLHHHHQHQHQLQHPPQQQPHPHQQLQHPLQQQQHLSAMDNHQQHQPHIHQQQQQQQQLPPTPQAS
HLVGGQQQQQQHPHHNHLAVDQDDPNPELVLALISRNRALEATIPKCGGCHELILDRFILKVLERTWHAKCLQCS
ECHGQLNDKCFARNGQLFCKEDFFKRYGTKCSACDMGIPPTQVVRRAQDNVYHLQCFLCAMCSRTLNTGDEFYLM
EDRKLICKRDYEEAKAKGLYLDGSLDGDQPNKRPRTTITAKQLETLKTAYNNSPKPARHVREQLSQDTGLDMRVV
QVWFQNRRAKEKRLKKDAGRTRWSQYFRSMKGNCSPRTDKFLDKDELKVDYDSFSHHDLSNDSYSTVNLGLDEGA
SPHSIRGSYMHGSSSPSQYPPSSRSPPPVGQGHTFGSYPDNIVYTNIDQAVGSSLHASKAHHRLHSSNNVSDLSN
DSSPDQGYPDFPPSPDSWLGDSGSTNTTSANNNANNNSSSSHNNNNNSSGGGSGGVSVSTAPNPSAPGVHY

CeLHX3 >sp|P20271|HM14_CAEEL Homeobox protein ceh-14 OS=Caenorhabditis elegans GN=ceh-14 PE=1 SV=3
MLGHNILTLGECDELDNHIVMCSTGLLSPQEDFSNVNAGHPNNEEAICSLCDKKIRDRFVSKVNGRCYHSSCLRC
STCKDELGATCFLREDSMYCRAHFYKKFGTKCSSCNEGIVPDHVVRKASNHVYHVECFQCFICKRSLETGEEFYL
IADDARLVCKDDYEQARDKHCNELEGDGSNKRPRTTISAKSLETLKQAYQTSSKPARHVREQLASETGLDMRVVQ
VWFQNRRAKEKRLKKDAGRRWKSSNRAESDSNSPIESINGQSPNYLYLDHPMDDGNESNYLFHSREQTPDKYYRN
ETPSTDPPPMHMTTPSVLTTNFSTPLSLSTNVYNLPPPESQLIPHMTPQYI

NvLHX3 >tr|C4B860|C4B860_NEMVE LIM homeobox protein 1 OS=Nematostella vectensis GN=lhx1 PE=2 SV=1
MVQQCAGCQLPIADKFLLKVLDGVWHAQCVQCSDCKCPLTERCFSREGKLFCKTDFYRRYGTKCSGCDQGISPND
MVRRAKHLVFHVDCFVCSYCKRQITTGDELYYIGDGSFICRDDYYHSHPTNLDDAIDEPKDLSYGLDEDLDAALA
SKRRGPRTTIKAKQLEALKSTFAATPKPSRNIREKLAQETGLNMRVIQVWFQNRRSKERRLKQSGGQTPTSRPSR
PSRRSRIARNNSDGSNESQQELQNSSHLNYQQDLQTPPSHYGNPSNFSEFYSQNSPVPDGISMNNSQLHPHPTNF
GSGQHMRRPASLPNNELAEGGGMATPQSLNLAGQSPGTGVFPAAMQHKSAIAGSDDVW

MlLHX3 >tr|H2BPZ7|H2BPZ7_MNELE Lhx3 OS=Mnemiopsis leidyi PE=2 SV=1
MTPAASISTPLILNSPPKSFSTSMPPMTLYDADSLATNGILQTSTSPGSMIISPPMVSSIPSSTIFSPLPVSYPP
VSGLQVGSLSGGPVLSTTPISTLPTSLPNSAVKFEQTSFHGDVTGAVVPRCAGCDQPITDRFILKVMEKNWHSAC
LKCHDCLAQLTDKCFSRGDFVYCKDDFYKRYGTKCAKCEKVIPPSQVVRRAGGHVFHMDCFVCIICSRTLNTGDE
FYFVDDNQLVCRSDYDNFKTQYANCTDETFTDELDLENQGIKRPRTTITAKQLETLKTAYENSPKPARHVREQLS
SETGLDMRVVQVWFQNRRAKEKRMKKEAPRNRWGQYLKNLRKNRPQARPVRAGPGTPLSGDVKLEQPEFPAPNSC
FPGPPGNDSMMDRNQIAPPYPGCPSPNSMIRLGLHSGDFMRTSVSNHAFVNGHIRPPFMGPAQPAPLFSSHFPGG
DVGNLGTNTAALGNAMRIAAGEEPYNEFPSAWLGPEEFDQRLPLPYSDQKPPFPAMI

AqLHX3 >tr|B1A9Y5|B1A9Y5_AMPQE Lim3 OS=Amphimedon queenslandica PE=2 SV=1
MSYQDINTHQTTSSPVVDPFQVPPSLPSIQTPQPSGMAMLVQPQSAAPSSLSLAQTPTQEYHQNLSSPSNQPQQI
QTSAPQAQDQNIAHVPFCAGCNTRIFDRFILRVQDKSWHAKCLRCSDCQCQLSDKCYSRSGQVYCKDDFSKRFGT
RCAGCQQPIPPTQVVRRAQENVYHLQCFACFICQRQLSTGDEFYLMDDRKLVCKADYEAAKARADGSQKRPRTTI
SQKQLDLLKTAYCVSPKPSRHVRQELSDKTGLDMRVVQVWFQNKRAKDKRTKKDDGSDEGAGEGEGRGGDGDFSV
STPTSATDDIQGQFILDPDIAQQGIDAPPPNPIGTTSA

TaLHX3 >TaLhx3/4 (53309) protein prediction[1]
MAIGNIDYKVPDFVITALYTLLSTFKCSIFGNTVNCSSRGYCMQAQGVATRIRNILQRFKSCRKDLKLLELDSRR
NELLCFKCQEEIRDRNIYEMDNQLWHGKCISCIDCGKNLEGKCFVKEESIYCSMHYYRRFGRKCQGCNLGILPDE
MVYRLHGSCYHINCLLCIVCSRQFKVGDKYYISDEGKPICKEDYDVAIMCSDDFQLHHPNLKRPRTSITQQQLKM
LNSVYRIKPRPSRITREMIATKLMEELTRLRILVRCVDTGCSLCTEIYTFNIVTPNCRTEFTICSIVMFTTPITN
*

Although there are some sequence similarities in an HD-like region (underlined), this similarity peters out partway through the sequence and appears to be too short to comprise a typical HD domain.

A

	pGBT9	pGAD10
1	LIM-7$_{LID}$	CEH-14$_{LIM1+2}$
2	LIM-7$_{LID}$	CEH-14$_{LIM1}$
3	LIM-7$_{LID}$	CEH-14$_{LIM2}$
4	CEH-14$_{LIM1+2}$	LIM-7$_{LID}$
5	CEH-14$_{LIM1}$	LIM-7$_{LID}$
6	CEH-14$_{LIM2}$	LIM-7$_{LID}$
7	LIM-7$_{LID}$	empty
8	empty	CEH-14$_{LIM1+2}$
9	empty	CEH-14$_{LIM1}$
10	empty	CEH-14$_{LIM2}$
11	empty	LIM-7$_{LID}$
12	CEH-14$_{LIM1+2}$	empty
13	CEH-14$_{LIM1}$	empty
14	CEH-14$_{LIM2}$	empty

B

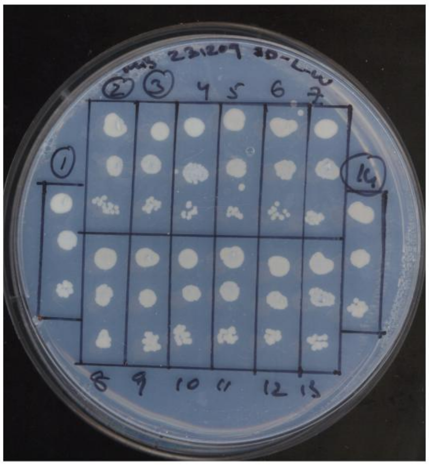

SD-L-W plates (transformation controls)

C

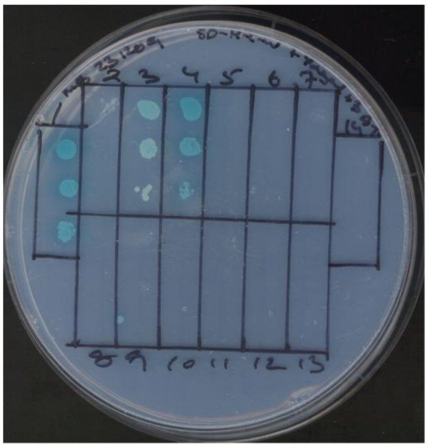

SD-L-W-H+3-AT+α-Gal plates
(Interaction-moderate stringency)

D

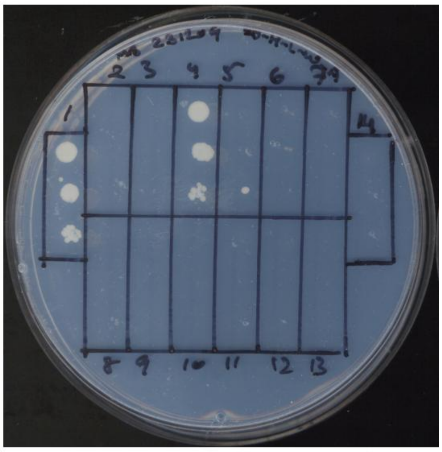

SD-H-L-W-H-A plates
(Interaction-high stringency)

Figure S2. Yeast two-hybrid data for LIM-7$_{LID}$: CEH-14$_{LIM1+2}$ (and individual LIM domain) interactions. **A** Key for plasmid combinations in each lane on all plates. **B** Transformation control data (SD-L-W). **C** Moderate stringency selection plate SD-L-W-H + 0.5 mM 3-AT + α-Gal. **D** Higher stringency selection plate. 2-μl aliquots of yeast from a 1:10 dilution series (*top*, $A_{600\ nm}$ = 0.2, 10^0; *middle*, 10^{-1}; *bottom*, 10^{-2})) were spotted on to each rectangular segment. The transformation control data show that yeast were successfully co-transformed. Under these selection conditions no growth was seen in negative control lanes (7–14). Lanes 1–6 from panels C and D are reproduced in Figure 1D.

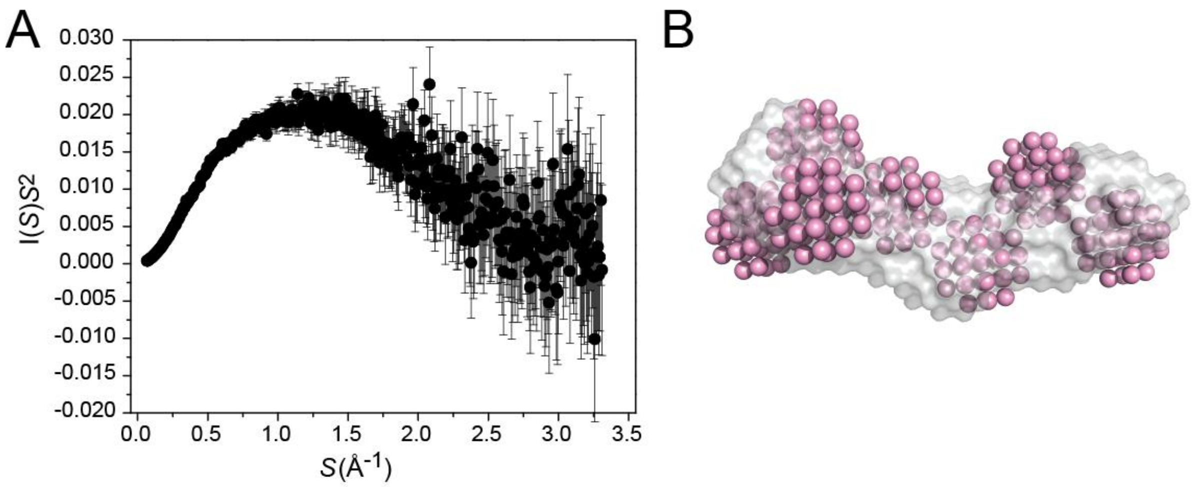

Figure S3 Additional assessment of foldedness and shape. A Kratky plot for CEH-14–LIM-7. A The desmeared data in Kratky format are noisy, but the bell shaped curve that plateaus at higher values of Q, indicate the protein is largely folded and does not exhibit large amounts of flexibility [2], consistent with the far-UV CD and NMR data. Kratky analysis does not readily distinguish between folded and partially unfolded proteins meaning that it is unlikely to report the foldness of residues (~10% in total) in the tether and ends of the domains[3]. **B** AMBIMETER output (pink spheres) overlaid with the shape restoration model of CEH-14–LIM-7 (white surface)

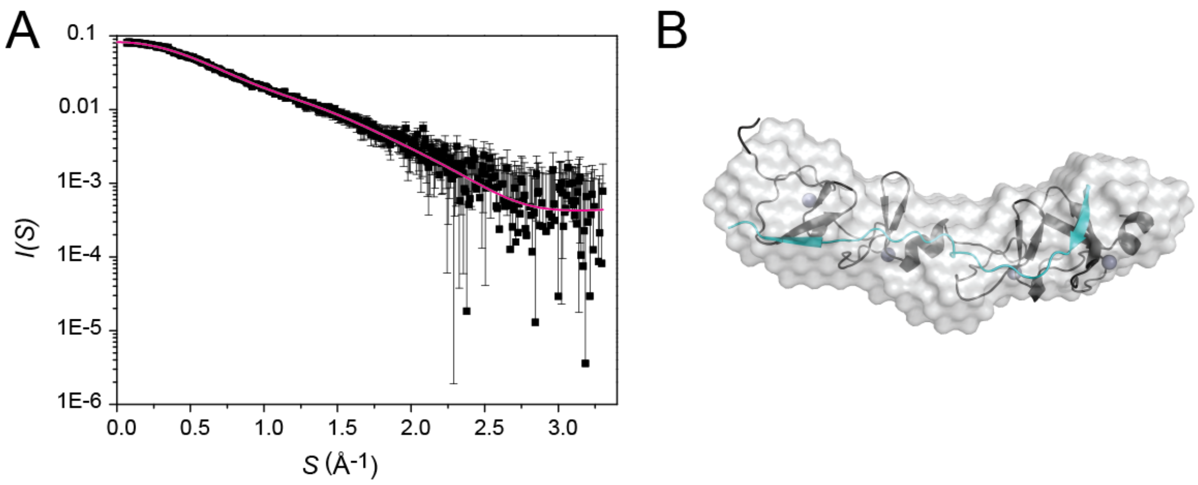

Fig S4 Comparison of CEH-14–LIM-7 and LHX3-ISL1. A) Buffer-corrected, desmeared SAXS scattering curve of CEH-14–LIM-7 (squares; 5.2 mg mL^{-1}) in 20 mM tris pH 8.0, 150 mM NaCl, 1 mM TCEP, overlaid with the theoretical scattering profiles of LHX3-ISL1 crystal structure (1RGT Chain B; magenta line) calculated by CRYSOL. **B)** Shape restoration model of CEH-14–LIM-7 (white surface) superimposed with the LHX3-ISL1 crystal structure (1RGT Chain B; black and cyan; zinc ions are shown as grey spheres).

Supplemental Data 5 – Summary of SwissModel Quality Assessment for CEH-14–LIM-7

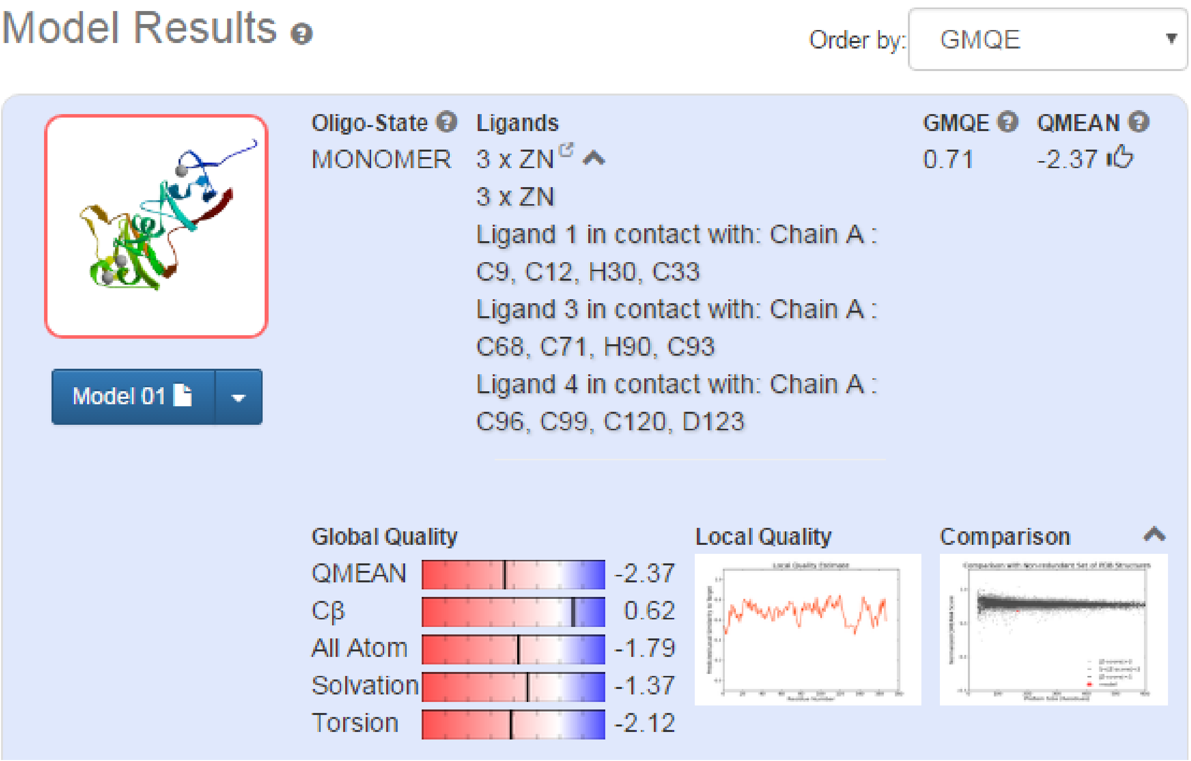

Per-residue QMEAN scores mapped onto the sequence and structure of CEH-14–LIM-7. Zinc-coordinating residues are indicated with rectangles, with sets of residues that coordinate the same group indicated by colour. Dotted lines indicate fully conserved residues, solid lines indicate non-conserved residues.

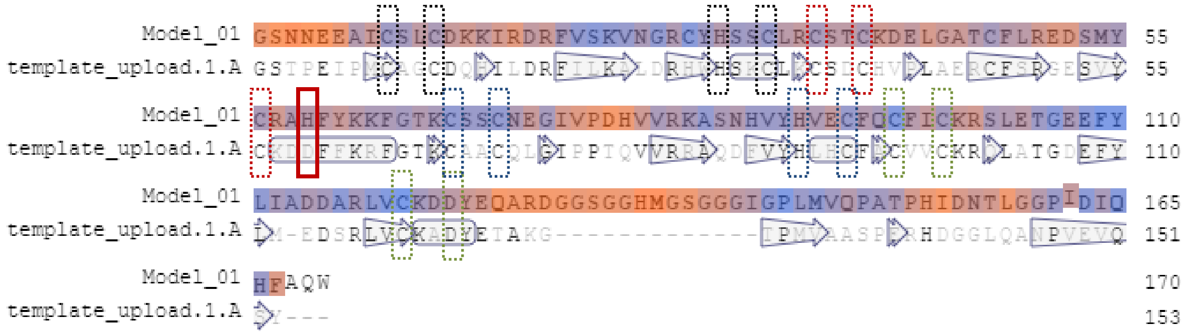

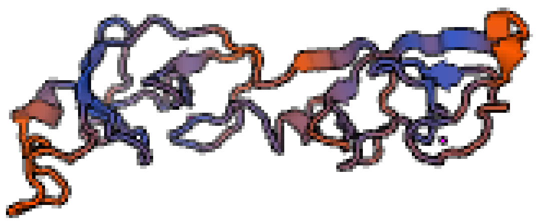

Note that three of the four zinc ions were correctly modelled into the structure, but the second zinc in the CEH-14$_{LIM1}$ was not because the zinc coordinating motif is not fully conserved

being CCCH in CEH-14 and CCCD in LHX3. SwissModel does not include ligands unless the binding residues are fully conserved. However, the coordination geometry of the putative is conserved and both motifs are found commonly in LIM domains.

Additional References

1 Srivastava, M. *et al.* Early evolution of the LIM homeobox gene family. *BMC Biol* 8, 4, (2010).

2 Glatter, O. & Kratky, O. *Small Angle X-ray Scattering*. (Academic Press, 1982).

3 Receveur-Brechot, V. & Durand, D. How random are intrinsically disordered proteins? A small angle scattering perspective. *Curr Protein Pept Sc* 13, 55-75, (2012).
